# Kinome‐Wide Synthetic Lethal Screen Identifies PANK4 as a Modulator of Temozolomide Resistance in Glioblastoma

**DOI:** 10.1002/advs.202306027

**Published:** 2024-02-14

**Authors:** Viviana Vella, Angeliki Ditsiou, Anna Chalari, Murat Eravci, Sarah K. Wooller, Teresa Gagliano, Cecilia Bani, Emanuela Kerschbamer, Christos Karakostas, Bin Xu, Yongchang Zhang, Frances M.G. Pearl, Gianluca Lopez, Ling Peng, Justin Stebbing, Apostolos Klinakis, Georgios Giamas

**Affiliations:** ^1^ Department of Biochemistry and Biomedicine School of Life Sciences University of Sussex, Falmer Brighton BN1 9QG UK; ^2^ Center of Basic Research Biomedical Research Foundation of the Academy of Athens Athens 11527 Greece; ^3^ School of Life Sciences Bioinformatics Group University of Sussex, Falmer Brighton BN1 9QG UK; ^4^ Department of Medicine University of Udine Udine 33100 Italy; ^5^ Cancer Center Renmin Hospital of Wuhan University Wuhan Hubei 430064 China; ^6^ Department of Medical Oncology Lung Cancer and Gastrointestinal Unit Hunan Cancer Hospital/The Affiliated Cancer Hospital of Xiangya School of Medicine Central South University Changsha Hunan 430064 China; ^7^ Division of Pathology Fondazione IRCCS Ca' Granda – Ospedale Maggiore Policlinico Milan 20122 Italy; ^8^ Department of Biomedical, Surgical and Dental Sciences University of Milan Milan 20122 Italy; ^9^ Department of Respiratory Disease Zhejiang Provincial People's Hospital Hangzhou Zhejiang 310003 China; ^10^ Department of Life Sciences Anglia Ruskin University East Road Cambridge CB1 1PT UK

**Keywords:** cellular detoxification, DUF89 domain, GBM, kinome‐wide RNAi screen, PANK4, TMT‐based quantitative proteomics, TMZ resistance

## Abstract

Temozolomide (TMZ) represents the cornerstone of therapy for glioblastoma (GBM). However, acquisition of resistance limits its therapeutic potential. The human kinome is an undisputable source of druggable targets, still, current knowledge remains confined to a limited fraction of it, with a multitude of under‐investigated proteins yet to be characterized. Here, following a kinome‐wide RNAi screen, pantothenate kinase 4 (PANK4) isuncovered as a modulator of TMZ resistance in GBM. Validation of PANK4 across various TMZ‐resistant GBM cell models, patient‐derived GBM cell lines, tissue samples, as well as in vivo studies, corroborates the potential translational significance of these findings. Moreover, PANK4 expression is induced during TMZ treatment, and its expression is associated with a worse clinical outcome. Furthermore, a Tandem Mass Tag (TMT)‐based quantitative proteomic approach, reveals that PANK4 abrogation leads to a significant downregulation of a host of proteins with central roles in cellular detoxification and cellular response to oxidative stress. More specifically, as cells undergo genotoxic stress during TMZ exposure, PANK4 depletion represents a crucial event that can lead to accumulation of intracellular reactive oxygen species (ROS) and subsequent cell death. Collectively, a previously unreported role for PANK4 in mediating therapeutic resistance to TMZ in GBM is unveiled.

## Graphical abstract

Resistance to temozolomide (TMZ) chemotherapy is a major hurdle for glioblastoma (GBM) treatment. Depletion of pantothenate kinase 4 (PANK4) is found to restore sensitivity to TMZ. By modulating a set of proteins with central roles in cellular detoxification, PANK4 abrogation can exacerbate the damage caused by TMZ, leading to critical accumulation of intracellular reactive oxygen species (ROS) and cell death.

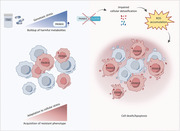

## 1 Introduction

Glioblastoma (GBM) is one of the most aggressive and lethal forms of primary brain and central nervous system (CNS) tumors, and it is essentially an incurable disease.^[^ ^1^, ^2^, ^3^ ^]^ The current mainstay of treatment for GBM patients is multimodal, as it consists of maximal surgical resection, followed by localized radiotherapy (RT) combined with concomitant and adjuvant chemotherapy with the alkylating agent temozolomide (TMZ).^[^ ^1^ ^]^ Acquisition of resistance to TMZ, is one of the main reasons why chemotherapy generally fails, posing a great challenge for the management of GBM patients.^[^ ^3^ ^]^

As in the case of other cancers,^[^ ^4^, ^5^ ^]^ aberrations in diverse core kinase‐signaling pathways have proved to be crucial for GBM initiation and progression and hence they have been intensively investigated.^[^ ^6^, ^7^ ^]^ Nevertheless, the human kinome encompasses a multitude of under‐investigated kinases with potential therapeutic relevance that may represent viable drug targets,^[^ ^8^, ^9^, ^10^ ^]^ albeit their role still remains unexplored in GBM therapeutic resistance. Intriguingly, pseudokinases represent a notable, yet poorly understood, fraction of the kinome which has garnered increased interest over the last few years.^[^ ^11^, ^12^, ^13^ ^]^ By signaling primarily through noncatalytic mechanisms, along with their unique structural features, pseudokinases play a critical role both in normal physiology and pathological conditions, including cancer.^[^ ^11^, ^12^, ^13^, ^14^, ^15^ ^]^

Pantothenate kinase 4 (PANK4) is an understudied but highly conserved protein.^[^ ^16^ ^]^ Unlike the other three members of the pantothenate kinases family (PANK1‐3), PANK4 has been characterized as a pseudokinase, due to mutations of specific residues that have rendered its kinase domain catalytically inactive.^[^ ^16^ ^]^ Interestingly, PANK4 also encompasses a C‐terminal phosphatase domain (DUF89) implicated in metabolite damage‐control processes.^[^ ^17^ ^]^ The limited number of existing studies have mainly attempted to examine its catalytic activity as well as its metabolic role in the biosynthesis of coenzyme A (CoA) and in the context of pantothenate kinase–associated neurodegeneration (PKAN) disorders.^[^ ^16^, ^17^, ^18^, ^19^ ^]^ Still, to date, its functional spectrum in physiological processes, cancer and other diseases, remains to be determined.

In this study, through a kinome‐wide RNAi screen, we identified PANK4 as a synthetic lethal partner of TMZ in drug‐resistant GBM cells and demonstrated that its depletion enhances the effect of TMZ, improving the response to TMZ therapy. More specifically, we showed that combined abrogation of PANK4 and TMZ treatment leads to attenuation of cell proliferation and clonogenicity, increased cell death in TMZ‐resistant GBM models, as well as decreased tumor growth in vivo. We also provide evidence that PANK4 expression is induced in response to TMZ treatment and increased PANK4 levels are associated with a worse clinical outcome. Moreover, by employing TMT‐based quantitative proteomics, we reveal a link between PANK4 and a set of proteins of the cellular detoxification system, consistent with its role in damage control,^[^ ^17^, ^20^ ^]^ and we uncover a previously unreported role of PANK4 in modulating the levels of reactive oxygen species (ROS) in TMZ‐resistant GBM cells. Furthermore, our data show that PANK4's ability to modulate sensitivity to TMZ treatment and ROS accumulation is dependent on its phosphatase activity. Our findings suggest that PANK4 depletion exacerbates the damage caused by TMZ by compromising the cellular detoxification mechanisms and shifting the balance towards an inefficient stress response, ultimately leading to cell death. In aggregate, we investigate the uncharacterized, yet highly attractive role of PANK4 in the context of TMZ resistance in GBM.

## 2 Results

### 2.1 Kinome‐Wide RNAi Screen Identifies PANK4 as a Synthetic Lethal Partner of TMZ

**Figure 1:**
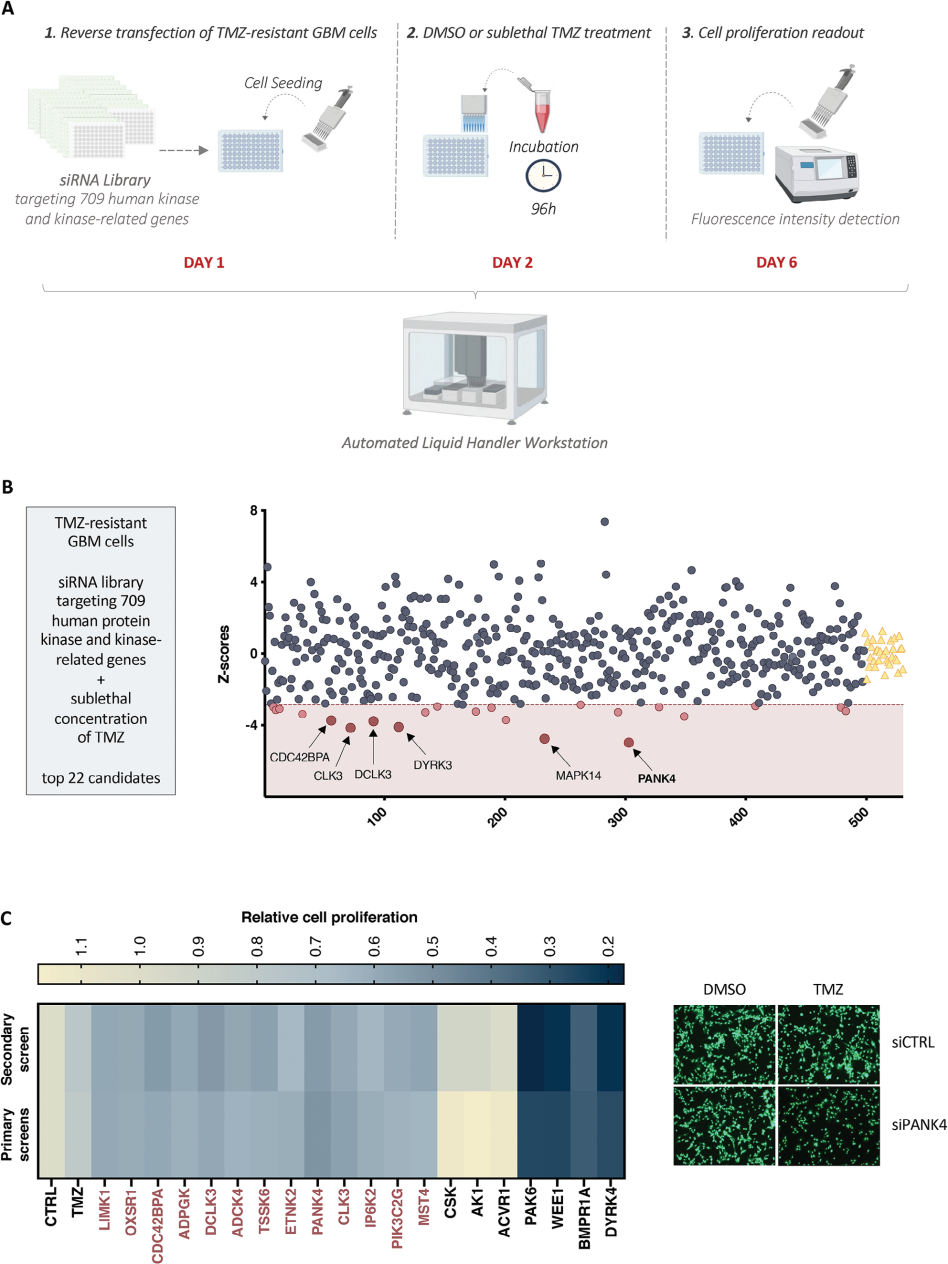
Kinome‐wide RNAi screen identifies PANK4 as a synthetic lethal partner of TMZ. A) Schematic representation of the synthetic lethal RNAi screen workflow. U87MG^Res^ cells were reverse‐transfected using an siRNA library targeting 709 human protein kinase and kinase‐related genes. Twenty‐four hours after transfection, cells were treated with either DMSO or a sublethal concentration of TMZ and incubated for 96 hours. On day 6, CyQuant reagent was added to the cells and fluorescence intensity was quantified as a measure of cell proliferation. Figure was created with BioRender.com. B) Z‐scores of synthetic lethal candidate genes generated from the RNAi primary screens. Red dots represent top‐candidates that significantly decreased cell proliferation in combination with TMZ (z‐score cut‐off: <−2.86). Yellow triangles represent TMZ controls. C) Left: Smaller‐scale secondary screen on U87MG^Res^ cells compared to our primary screens (average of two independent screens). The heatmap displays the combined effect of gene knockdown and TMZ treatment on cell proliferation calculated for each of the indicated kinases. Blue and yellow denote either reduction or increase in cell proliferation, respectively. A total of 20 protein kinase genes were randomly selected for validation. Protein kinases belonging to the top 22 candidates are highlighted in red (= 13), including PANK4. Right: Representative images of U87MG^Res^ cells labelled with CyQuant green fluorescent nucleic acid stain, demonstrating the antiproliferative effect of PANK4 knockdown and sublethal TMZ treatment. Magnification, 10×.

To explore the role of protein kinase signaling in temozolomide (TMZ) resistance, we established the experimental pipeline shown in **Figure** **1**A. A kinome‐wide RNAi screen using a small interfering RNA (siRNA) library was performed. Briefly, TMZ‐resistant U87MG cells derived from the “Resistant Cancer Cell Line (RCCL) collection” and generated by chronic exposure to the drug (from now on referred to as U87MG^Res^),^[^ ^21^ ^]^ were transfected with siRNA pools targeting each of the 709 human protein kinase and kinase‐related genes (Day 1) and treated with a sublethal dose of TMZ or DMSO (Day 2). Subsequently, differences in cell proliferation following siRNA knockdown and TMZ treatment were assessed (Day 6).

The primary screen was performed twice and the biological reproducibility of the two screen experiments was evaluated showing good statistical correlation (Figure S1A, Supporting Information). As the purpose of this study was to identify drug/siRNA combinations that resulted in a synthetic lethal effect, a sublethal concentration of TMZ able to inhibit cell proliferation by 20% (IC_20_) was used for this screen (Figure S1B, Supporting Information). Similarly, as for the data generated from the primary screens (Table S1, Supporting Information), all gene candidates having an independent effect of >20% on cell proliferation were excluded from further analysis. This way, we sought out to uncover targets that are critical for cell proliferation only in the presence of the drug, and therefore display a synthetic lethal effect with TMZ. Under these conditions, we unveiled 22 statistically significant top‐ranking genes as potential targets in our glioblastoma (GBM) cell model, with pantothenate kinase 4 (PANK4) being the most effective hit (Figure 1B, Figure S1C,D, Supporting Information).

To further assess the biological reproducibility of our results, we implemented a secondary screen on the same TMZ‐resistant GBM cell model. A number of randomly selected kinases was used for confirmation. Consistent effects were observed across the independent screens, proving the ability of the selected kinases to reproduce the synthetic lethal phenotype observed in our primary screens (Figure 1C).

Taken together, based on its highest z‐score rank and considering its largely unexplored, yet intriguing role in cancer, we focused on PANK4 in order to investigate its potential role as chemosensitizer of TMZ in GBM.

### 2.2 PANK4 Knockdown Enhances the Chemosensitivity of TMZ‐Resistant GBM Cells

The prospect of PANK4 as a target for re‐sensitization to TMZ treatment was assessed across additional TMZ‐resistant GBM cells.^[^ ^21^, ^22^ ^]^ These included drug‐resistant T98G and U251 cells (from now on referred to as T98G^Res^ and U251^Res^ respectively) that were established following continuous exposure of their parental counterparts to increasing TMZ concentrations,^[^ ^21^, ^22^ ^]^ as well as the inherently TMZ‐resistant T98G cell line (from now on referred to as T98G^Par^).

Variable PANK4 protein expression levels and different drug susceptibility profiles were observed across the tested cell lines, therefore a sublethal concentration of TMZ able to inhibit cell proliferation by 20% was determined for each cell line (IC_20_) (**Figure** **2**A). All our drug‐resistant cell models were then assessed under the same conditions used in the original screen (Figure 1A). Combined PANK4 silencing and treatment with sublethal doses of TMZ were able to potentiate the effect of TMZ in a synergistic manner, leading to a decrease in cell proliferation (Figure 2B,C). Moreover, a significant impairment of cell proliferation was observed upon silencing of PANK4 and treatment with increasing concentrations of TMZ (Figure 2D). Similar antiproliferative effects were also detected in patient‐derived GBM cell lines obtained from the “Human Glioblastoma Cell Culture” (HGCC) biobank,^[^ ^23^ ^]^ where knockdown of PANK4 followed by TMZ treatment led to an improved response to the drug (Figure 2E).

Figure 2PANK4 knockdown enhances the chemosensitivity of TMZ‐resistant GBM cells. A) Left: Western blot of PANK4 expression levels in the indicated TMZ‐resistant GBM cell lines. Tubulin was used as loading control. Right: The same cancer cell lines were treated with increasing concentrations of TMZ and their dose response curves are shown. Cell proliferation was assessed at 96 hours and sublethal concentrations of TMZ were determined for all cell lines, following calculation of IC_20_ values using the GraphPad Prism 9 software. B) Cells were transfected with either siCTRL or siPANK4 and treated with sublethal concentrations of TMZ or DMSO after 24 hours. Cell proliferation was evaluated at 96 hours. The Cooperativity Index (CI) is shown for each cell line. PANK4 knockdown was confirmed by western blotting. Tubulin was used as loading control. C) Representative images of the proliferation assays shown in B. Magnification, 10×. Scale bar, 400 µm. D) Cells were transfected with either siCTRL or siPANK4 for 24 hours and treated with increasing concentrations of TMZ or DMSO, as indicated. Cell proliferation was assessed after 96 hours. E) Left: Western blot of PANK4 expression levels in the indicated patient‐derived GBM cell lines. Tubulin was used as loading control. Right: Cell proliferation of the same cells, following transfection with either siCTRL or siPANK4 and treatment with sublethal concentrations of TMZ or DMSO, as described in B. F) Control and stably PANK4‐depleted T98G^Res^ cells (shCTRL/shPANK4) were transfected with either pCMV6 or pCMV6‐PANK4. After 24 hours, cells were treated with DMSO or TMZ. The effect of PANK4 overexpression on cell proliferation was assessed after 96 hours. Representative images and western blot analysis of PANK4 expression are shown. Tubulin was used as loading control. All data are presented as mean ± SEM. Each experiment was conducted at least three times. Statistical analysis was performed using two‐way ANOVA (B, D, E, F); asterisks (*) designate significant differences between conditions indicated with brackets (B, E, F) or compared with the corresponding DMSO‐treated siRNA controls D) (*p*‐values: *p < 0.05, **p < 0.01, ***p < 0.001, and ****p < 0.0001).

**Figure:**
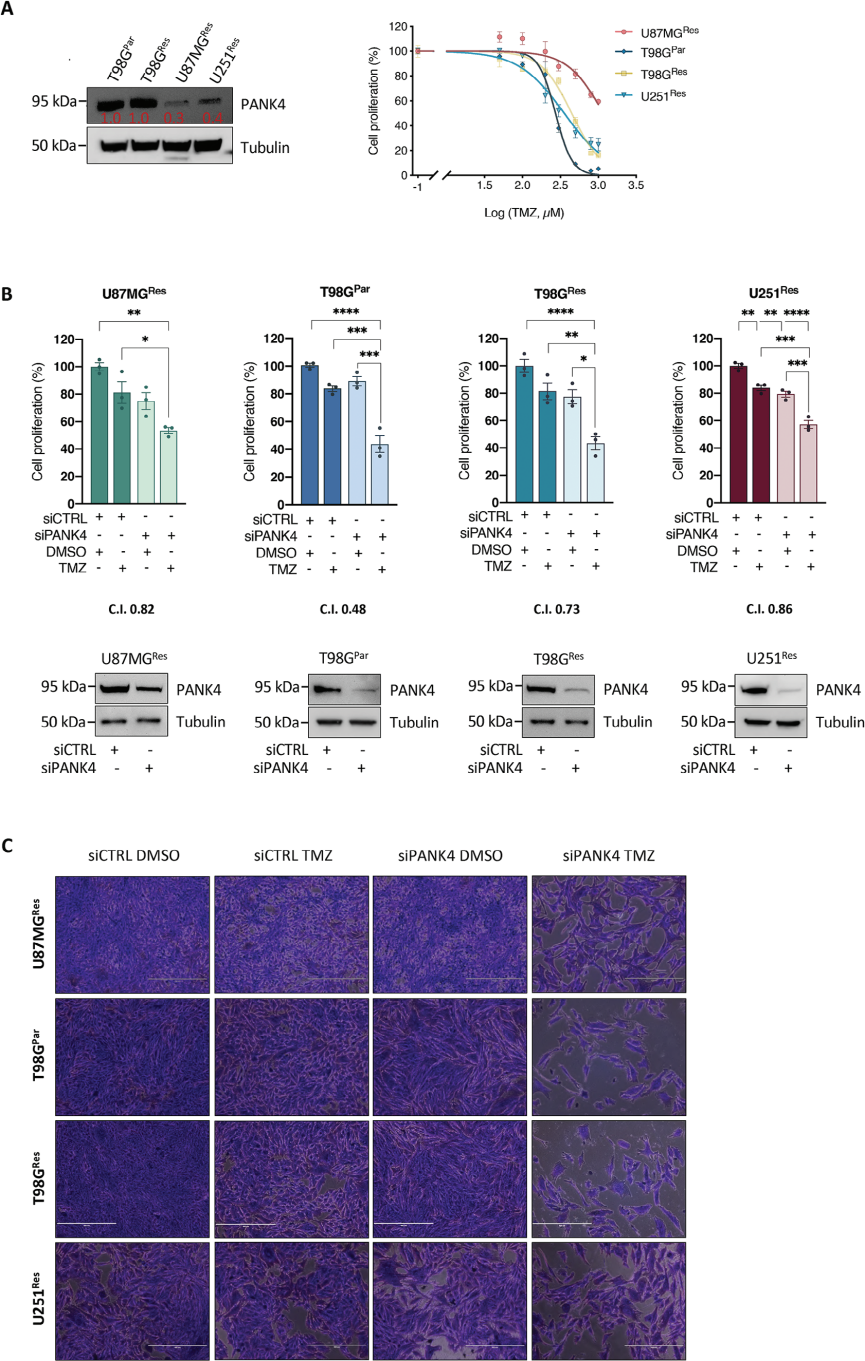

**Figure:**
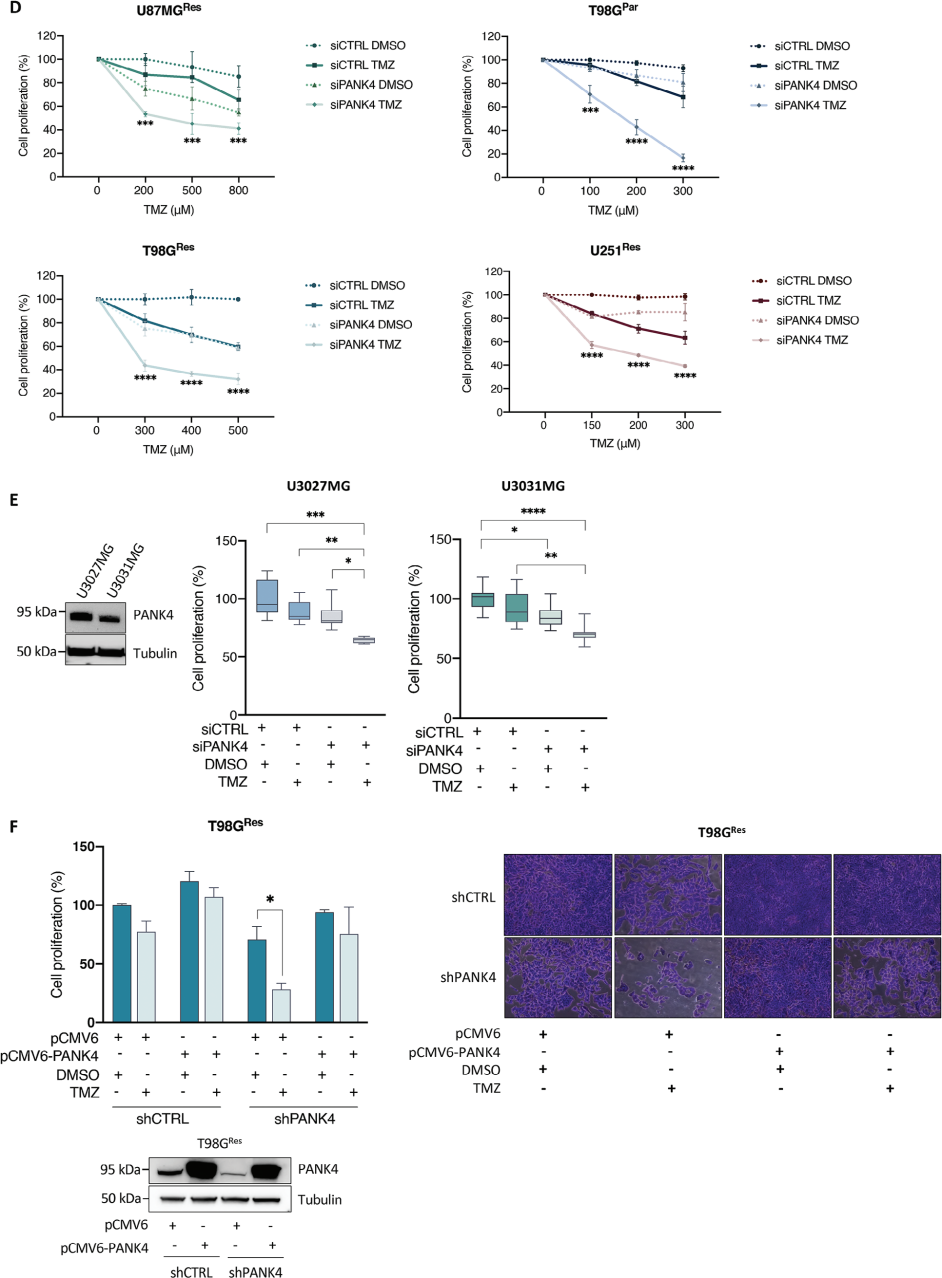

To corroborate our findings, we next assessed whether the observed phenotype could be rescued through gain‐of‐function experiments in stably PANK4‐depleted T98G^Res^ cells. As shown in Figure 2F, re‐expression of PANK4 abrogated sensitivity to TMZ treatment, restoring resistance to the drug. All PANK4‐targeting RNAi tools were validated for their ability to provide efficient and sustained PANK4 knockdown as shown in Figure S2A–E (Supporting Information).

Altogether, our results support that combined silencing of PANK4 and TMZ treatment significantly impede proliferation of drug‐resistant GBM cells, further emphasizing the chemo‐sensitizing potential of PANK4 depletion.

### 2.3 PANK4 Depletion Potentiates TMZ Cytotoxicity by Reducing the Clonogenic Potential of Resistant GBM Cell Lines

**Figure 3:**
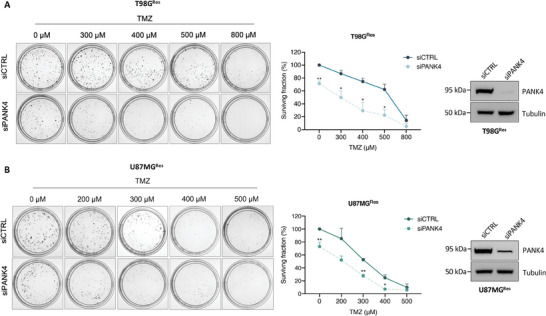
PANK4 depletion potentiates TMZ cytotoxicity by reducing the clonogenic potential of resistant GBM cell lines. A) Representative images of colony formation assays in control or PANK4‐depleted T98G^Res^ and B) U87MG^Res^ cells following treatment with TMZ, as indicated. Colonies were quantified and results show the percentage of colonies formed after treatment with the indicated concentrations of the drug (surviving fraction), corrected according to the plating efficiencies of the corresponding controls. PANK4 silencing was confirmed by western blot. Tubulin was used as loading control. Data are shown as mean ± SEM (n = 3 biological replicates). Significance was determined using unpaired Student's *t*‐test; asterisks (*) designate significant differences compared with the corresponding TMZ‐treated siRNA controls (*p*‐values: *p < 0.05, **p < 0.01).

To test whether silencing of PANK4 also has a long‐term chemo‐sensitizing effect in our resistant GBM cell models following exposure to TMZ, we conducted clonogenic cell survival assays. Our results showed that the colony‐forming ability of T98G^Res^ and U87MG^Res^ cells treated with TMZ was significantly impaired following silencing of PANK4 (**Figure** **3**A,B). The observed decrease in clonogenicity further supports our notion that PANK4 depletion enhances TMZ cytotoxicity, rendering TMZ‐resistant GBM cells more susceptible to the treatment.

### 2.4 PANK4 Downregulation Induces Apoptotic Cell Death upon TMZ Treatment

**Figure 4:**
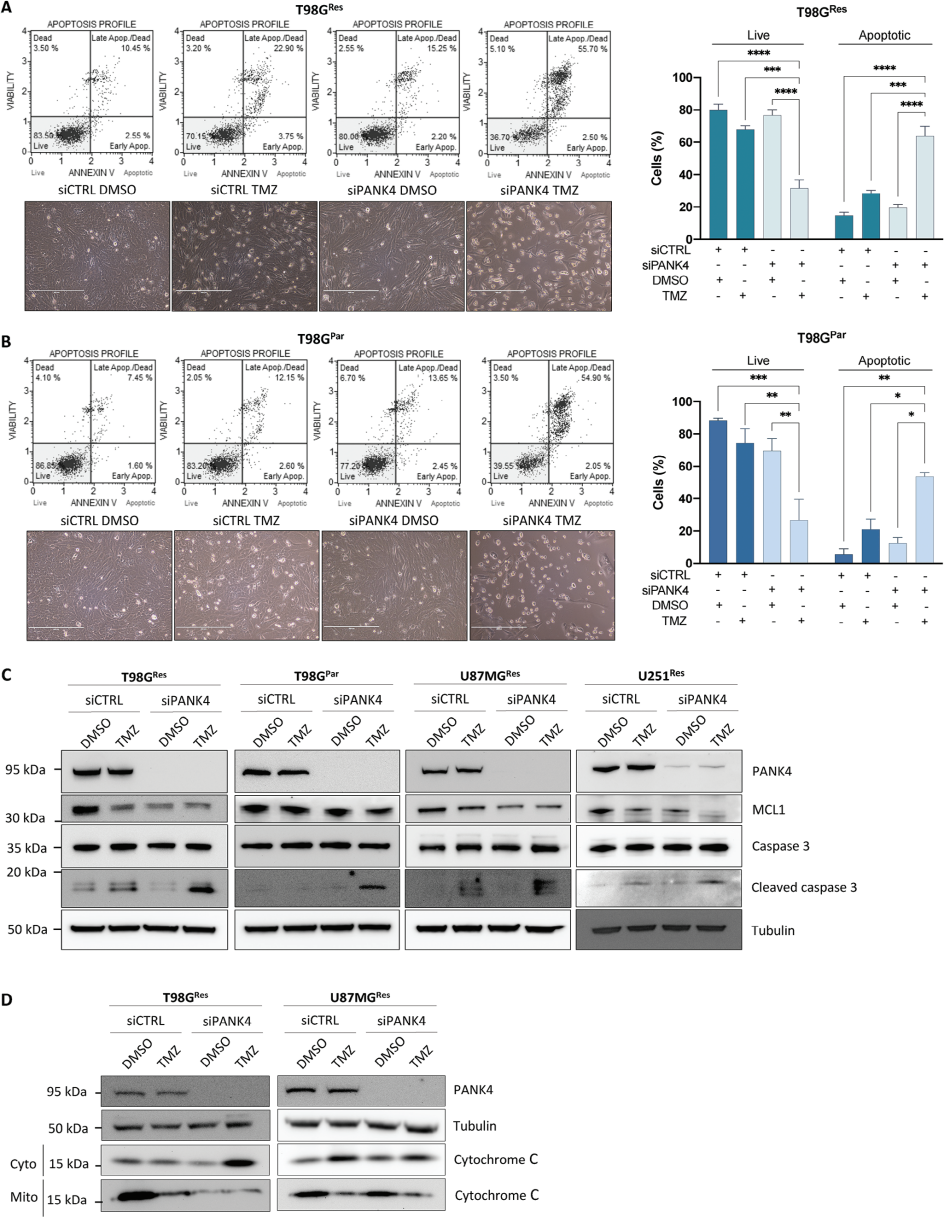
PANK4 downregulation induces apoptotic cell death upon TMZ treatment. A) T98G^Res^ and B) T98G^Par^ cells were transfected with either siCTRL or siPANK4. Twenty‐four hours after transfection, treatments with sublethal concentrations of TMZ were performed and the percentages of apoptotic cells were determined following annexin V and 7‐AAD staining (96 hours). Representative plots are shown. All data are presented as mean ± SEM. Each experiment was conducted at least three times. Statistical analysis was performed using two‐way ANOVA; asterisks (*) designate significant differences between conditions indicated with brackets (*p*‐values: *p < 0.05, **p < 0.01, ***p < 0.001, and ****p < 0.0001). C) Western blots showing expression of PANK4, MCL1, caspase 3, and cleaved caspase 3 in control and PANK4‐depleted T98G^Res^, T98G^Par^, U87MG^Res^, and U251^Res^ cells treated with DMSO or TMZ for 96 hours. Tubulin was used as loading control. D) Representative western blot showing expression of PANK4 and cytochrome c in control and PANK4‐depleted T98G^Res^ and U87MG^Res^ cells treated with DMSO or TMZ for 96 hours. Tubulin was used as loading control. Cytosolic and mitochondrial fractions are shown: Cyto, cytosol; Mito, mitochondria.

The phenotype resulting from simultaneous PANK4 knockdown and TMZ treatment was contradistinguished by a significant decrease in cell proliferation and induction of cell death. To examine this effect, we measured the apoptosis levels using annexin V and 7‐AAD (7‐aminoactinomycin D) staining. Neither treatment with sublethal concentrations of TMZ or silencing of PANK4 alone were able to significantly affect cell viability or induce apoptosis. However, a pronounced reduction in cell viability and a robust increase in the percentage of apoptotic cells was observed following combined PANK4 depletion and TMZ treatment (**Figure** **4**A,B). Moreover, assessment of a set of apoptotic markers confirmed that TMZ treatment combined with PANK4 knockdown triggered the activation of apoptotic signaling pathways, by downregulating MCL1 (myeloid cell leukemia‐1)^[^ ^24^ ^]^ and activating caspase 3^[^ ^25^ ^]^ (Figure 4C). Furthermore, PANK4 depletion in TMZ‐treated cells resulted in high levels of cytochrome c release from the mitochondria to the cytosol (Figure 4D). This eventually culminated into the apoptosis of GBM cells, as demonstrated by the substantial upregulation of cleaved caspase 3. In summary, these data indicate that PANK4 silencing enhances cell death by activating apoptotic signaling pathways following TMZ treatment.

### 2.5 Abrogation of PANK4 Sensitizes Chemo‐Resistant GBM Tumors to TMZ Treatment In Vivo

Our aforementioned cell‐based assays demonstrated that while silencing of PANK4 displays modest phenotypic effects, PANK4 knockdown in combination with TMZ treatment significantly potentiates TMZ cytotoxicity, sensitizing TMZ‐resistant GBM cells to the drug. To validate our findings in vivo, we established the experimental pipeline summarized in **Figure** **5**A. First, we assessed the effect of PANK4 silencing and confirmed its effective knockdown. Consistent with our in vitro results, no significant changes were observed on tumor growth in the absence of the drug (Figure 5B). Following establishment of a sublethal TMZ concentration (IC_20_) in vivo (Figure 5C,D), we evaluated the effect of TMZ treatment either alone or in combination with PANK4 knockdown. While treatment of mice with sublethal doses of TMZ did not significantly affect tumor growth, susceptibility to the drug was significantly improved following PANK4 silencing, as shown by the pronounced reduction in tumor growth (Figure 5E,F, Table S2, Supporting Information). Moreover, in support of our in vitro results, immunohistochemical (IHC) analysis showed no significant changes in Ki‐67 expression upon PANK4 silencing or TMZ treatment alone (Figure 5G,H). However, a considerable decrease in proliferation was detected in harvested tumors following PANK4 depletion and TMZ treatment (Figure 5H). We next assessed the induction of cell apoptosis upon combined abrogation of PANK4 and TMZ treatment in vivo. Consistent with our in vitro results, we saw an increased caspase 3 activation in TMZ‐treated samples following PANK4 knockdown (Figure S3A, Supporting Information). Moreover, IHC analysis of cleaved caspase 3 in tumor tissues following PANK4 knockdown and TMZ treatment further confirmed a pronounced upregulation of cleaved caspase 3 compared to control samples treated with TMZ alone (Figure S3B, Supporting Information). In line with our in vitro findings, PANK4 depletion positively modulates sensitivity to TMZ in vivo and renders chemo‐resistant tumors more vulnerable to the drug, further highlighting PANK4's role in TMZ resistance.

Figure 5Abrogation of PANK4 sensitises chemoresistant GBM tumors to TMZ treatment in vivo. A) Schematic representation of the experimental design of our in vivo study. Four mouse cohorts were established: siCTRL DMSO, siPANK4 DMSO, siCTRL TMZ, and siPANK4 TMZ (n = 6 mice per group). Figure was created with BioRender.com. B) Effect of PANK4 knockdown on tumor growth of mice carrying T98G^Res^ xenografts (n = 6 mice per group). Western blot and densitometric analysis of PANK4 expression in tumor lysates from three distinct tumors is shown confirming PANK4 knockdown efficiency. GAPDH was used as loading control. Error bars represent ± SEM. Significance was calculated using unpaired Student's *t*‐test; asterisks (*) designate significant differences between conditions indicated with brackets (ns, not significant; *p < 0.05). C) T98G^Res^ xenograft mice were treated with either vehicle control or TMZ at the indicated concentrations (n = 6 mice per group) and D) the in vivo sublethal dose of TMZ was subsequently determined using the GraphPad Prism 9 software. (E and F) Effect of combined PANK4 knockdown and treatment with the sublethal dose of TMZ on tumor growth of T98G^Res^ xenograft mice. (G) Immunohistochemical (IHC) evaluation of Ki‐67 expression in tumor sections from T98G^Res^ xenograft mice following PANK4 knockdown or H) treated with the sublethal dose of TMZ alone or following PANK4 depletion. Fold change of Ki‐67‐positive cells versus the total number of cells is shown. Data represent average of four independent samples per cohort, in duplicate. Representative images of Ki‐67 immunohistochemical staining in harvested tumors from each cohort are presented. Original magnification, 20x. Scale bar, 50 µm. E–H) Results are expressed as mean ± SEM. Significance was calculated using unpaired Student's *t*‐test; asterisks (*) designate significant differences between conditions indicated with brackets (ns, not significant; *p < 0.05, **p < 0.01).

**Figure:**
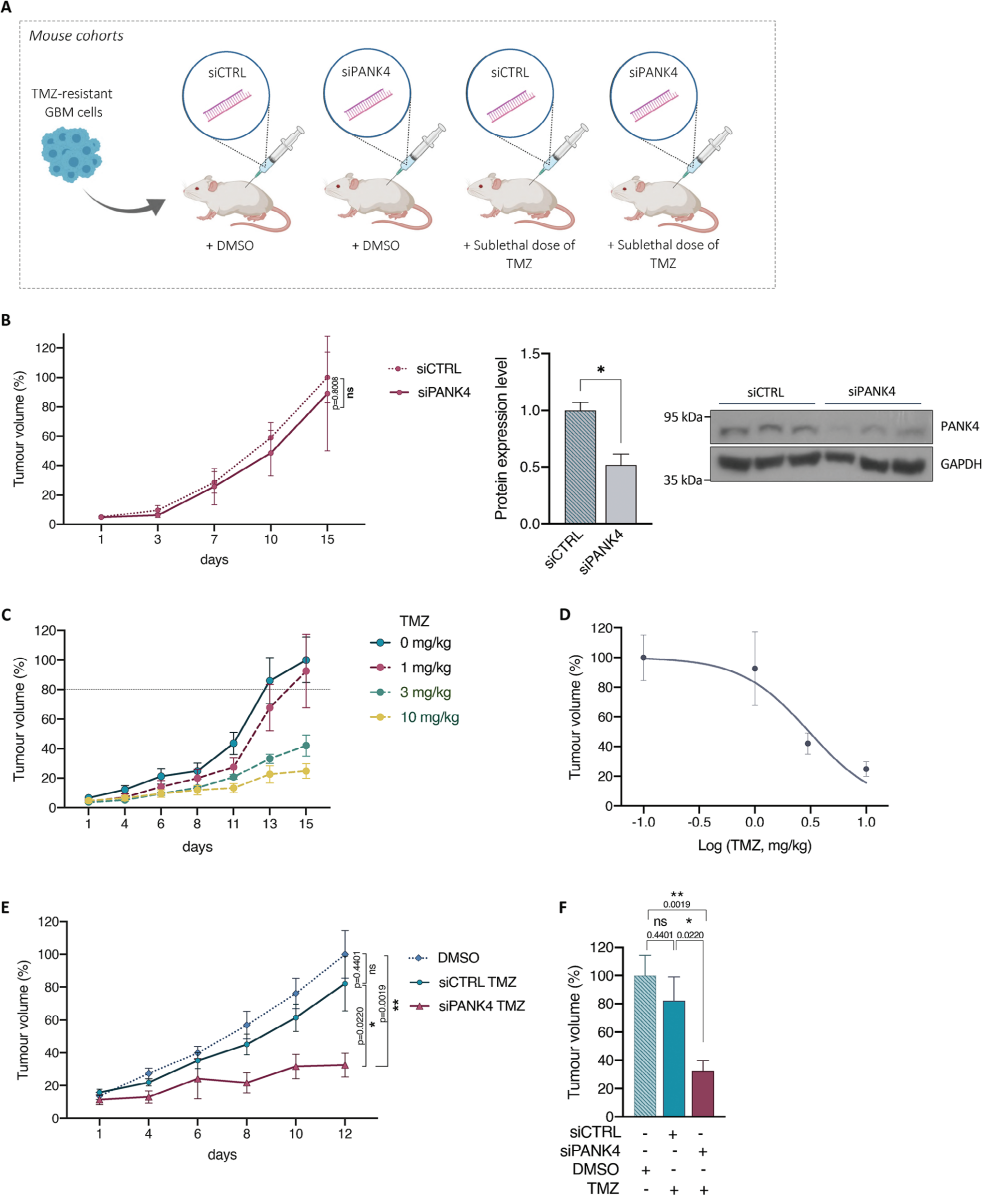

**Figure:**
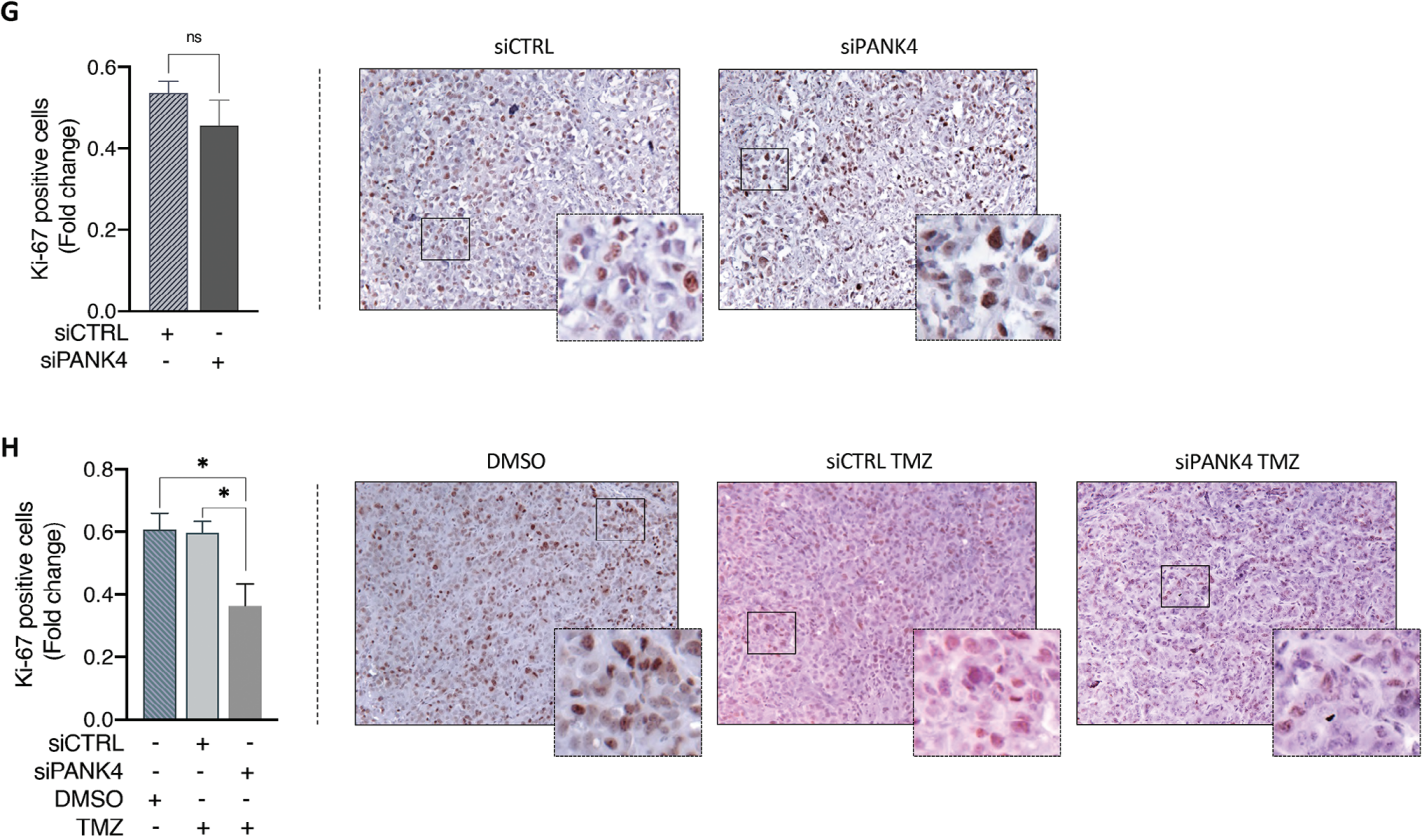

### 2.6 PANK4 Expression Profile in GBM Patient Cohorts and Its Association with TMZ Resistance

**Figure 6:**
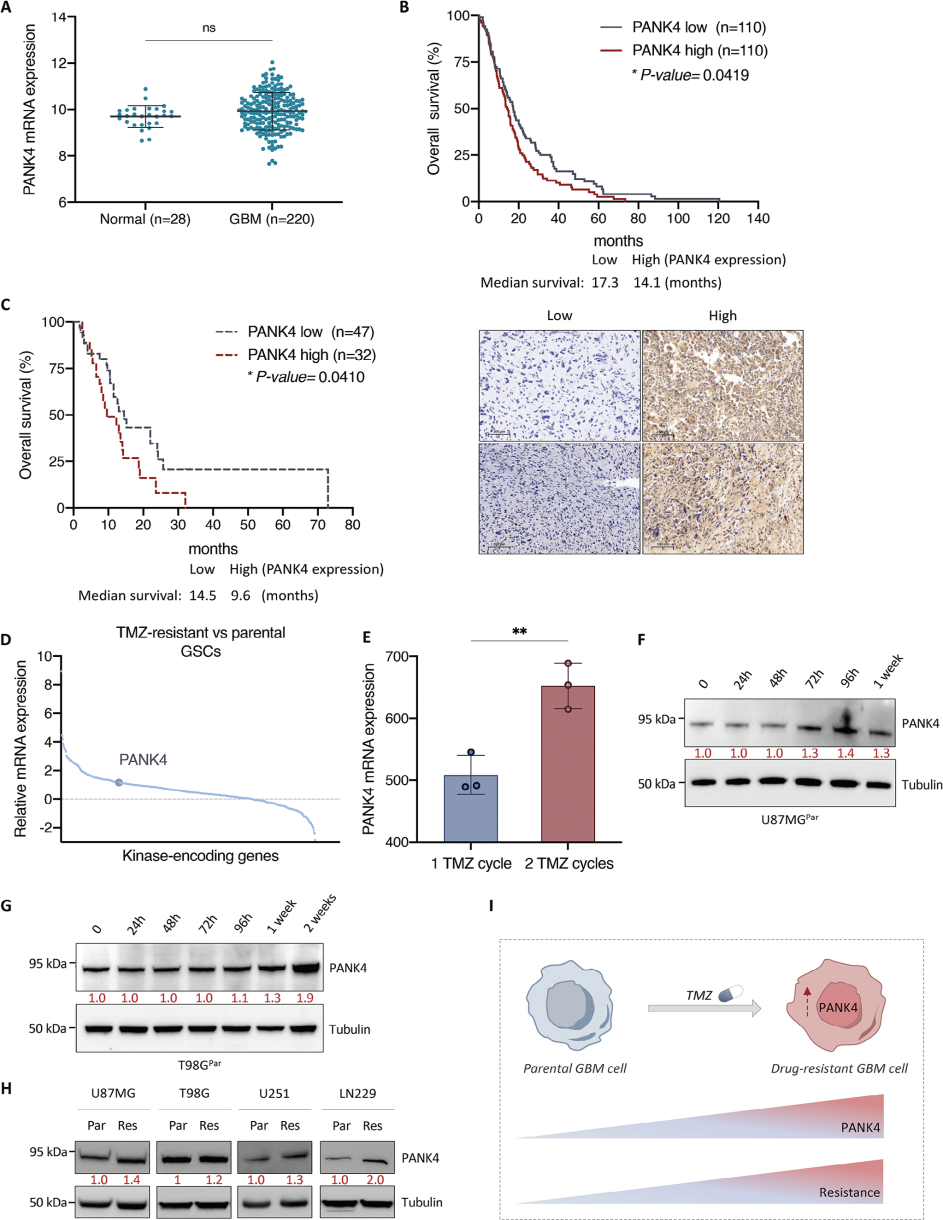
PANK4 expression profile in GBM patient cohorts and its association with TMZ resistance. A) Relative PANK4 mRNA expression in normal versus GBM tissue samples. Data derived from the Rembrandt brain cancer dataset (https://gdoc.georgetown.edu).^[^ ^26^ ^]^ B) Kaplan–Meier survival analysis showing the association between PANK4 mRNA expression and overall survival (OS) of GBM patients within the Rembrandt database and C) Kaplan–Meier plot showing the association between PANK4 protein expression, assessed by immunohistochemical analysis (IHC), and OS of GBM IDH‐wildtype patients. Statistical significance was evaluated by log‐rank analysis. Representative images of low and high immunohistochemical staining intensity of PANK4 protein expression in GBM tissue sections are shown alongside. Scale bar, 100 µm. D) Analysis for differentially expressed kinases (DEKs) in TMZ‐resistant versus parental GBM stem cells (GSCs). PANK4 was upregulated in the TMZ‐resistant group. Figure was generated based on data from the Gene Expression Omnibus (GEO; www.ncbi.nlm.nih.gov/geo, accession number: GSE68029).^[^ ^28^, ^29^ ^]^ E) Analysis of PANK4 mRNA expression in TMZ‐resistant GSCs that survived either one or two cycles of TMZ. Data from the GEO (www.ncbi.nlm.nih.gov/geo, accession number: GSE68029).^[^ ^28^ ^]^ F) Representative western blots of PANK4 expression in U87MG^Par^ and G) T98G^Par^ cells, following treatment with the TMZ concentrations used to generate their resistant counterparts, for the indicated time points. Tubulin was used as loading control. H) Representative western blots of PANK4 expression levels of in parental versus TMZ‐resistant cell lines (U87MG, T98G, U251, LN229). Tubulin was used as loading control. F–H) Densitometric analysis of PANK4 expression is shown. I) Schematic model illustrating the involvement of PANK4 in TMZ resistance in GBM. Figure was created with BioRender.com. A, E) Error bars represent ± SD. Significance was determined by unpaired Student's *t*‐test (ns, not significant; **p < 0.01).

To evaluate the clinical relevance of PANK4 expression in GBM tumors, we analyzed the REMBRANDT (Repository of Molecular Brain Neoplasia Data) dataset.^[^ ^26^ ^]^ No significant differences in PANK4 mRNA levels were observed between GBM tumors and normal brain tissue (**Figure** **6**A). Nevertheless, in line with previous studies on acute myeloid leukemia (AML),^[^ ^27^ ^]^ Kaplan–Meier survival analysis revealed that increased PANK4 mRNA expression is associated with decreased overall survival (OS) of patients suffering from GBM (Figure 6B). In agreement with these data, our IHC analysis performed on a cohort of GBM patients further suggested a link between reduced PANK4 expression levels and improved OS (Figure 6C; Figure S4A, Supporting Information).

To further explore the association between PANK4 expression and TMZ resistance, we used the Gene Expression Omnibus database (GEO; accession number: GSE68029) and examined kinases that were previously reported to be differentially expressed in TMZ‐resistant and parental GBM stem cells (GSCs).^[^ ^28^, ^29^ ^]^ Intriguingly, PANK4 was found to be upregulated in the TMZ‐resistant group compared with parental cells that were sensitive to the drug. (Figure 6D). Further analysis of the TMZ‐resistant group revealed significantly higher PANK4 mRNA levels in TMZ‐resistant GSCs that survived two cycles of TMZ treatment over GSCs that survived one cycle of TMZ treatment only (Figure 6E).^[^ ^28^ ^]^

Therefore, we next sought to determine whether PANK4 expression may be induced during TMZ treatment. To assess this, U87MG and T98G parental cells (referred to as U87MG^Par^ and T98G^Par^ respectively) were treated with TMZ at different time points. Treatment with the drug triggered a progressive increase both in PANK4 mRNA (Figure S4B,C, Supporting Information) and protein expression levels (Figure 6F,G). In addition, our TMZ‐resistant cell lines displayed notably higher PANK4 protein levels than their parental counterparts (Figure 6H).

Combined, these findings support that PANK4 expression is prompted in response to TMZ treatment and elevated PANK4 levels are maintained in cells that have acquired a resistant phenotype (Figure 6I). This suggests that PANK4 expression could be induced during TMZ chemotherapy, implying a potential requirement for PANK4 in response to TMZ.

### 2.7 TMT‐Based Proteomic Analysis Reveals Reduced Cell Detoxification Response upon PANK4 Knockdown

**Figure 8:**
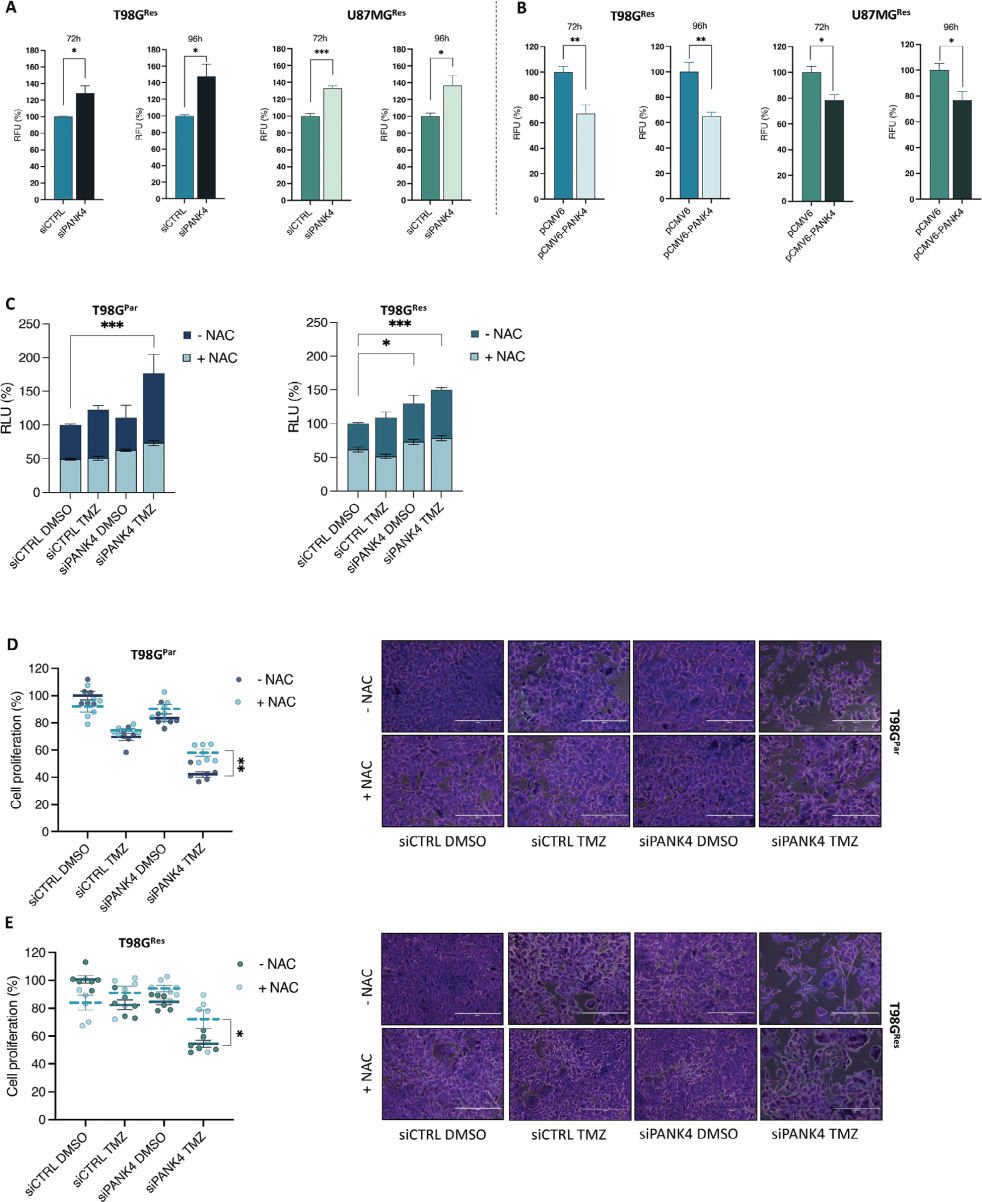
Modulation of PANK4 affects intracellular ROS levels of resistant GBM cells. A) T98G^Res^ cells and U87MG^Res^ cells were transfected with either siCTRL or siPANK4 and ROS levels were evaluated at 72 and 96 hours, as indicated. B) T98G^Res^ cells and U87MG^Res^ cells were transfected with either pCMV6 or pCMV6‐PANK4 and ROS levels were assessed at 72 and 96 hours. C) T98G^Par^ and T98G^Res^ cells were transfected with either siCTRL or siPANK4 and treated with sublethal concentrations of DMSO or TMZ after 24 hours, in the presence or absence of NAC (5 mm). ROS levels were evaluated at 96 hours. D) T98G^Par^ and E) T98G^Res^ cells were transfected with either siCTRL or siPANK4 and treated with sublethal concentrations of DMSO or TMZ after 24 hours, in the presence or absence of NAC (5 mm). Cell proliferation was evaluated at 96 hours. Representative images of the proliferation assays are presented alongside. Magnification, 10×. Scale bar, 400 µm.

To further explore the contribution of PANK4 to TMZ resistance, we performed a comprehensive proteomic characterization of TMZ‐resistant GBM cells using a quantitative Tandem Mass Tagging (TMT)‐based proteomic approach (Table S3, Supporting Information), as summarized in **Figure** **7**A. Given the largely unexplored functions of PANK4, we initially focused on changes in protein abundance following PANK4 silencing. Global proteomic analysis resulted in the identification of 6756 peptides of which 1005 were significantly altered after PANK4 knockdown (*p* < 0.05) (Figure 7B; Figure S5A, Supporting Information). Interestingly, Gene Ontology (GO) over‐representation analysis of statistically significant deregulated proteins uncovered a marked downregulation of biological processes (BP) linked to “cellular detoxification”, “cellular response to toxic substance”, and “detoxification”; these being among the top 20 downregulated biological processes impacted by PANK4 depletion (Figure 7C). Notably, this is in line with PANK4's previously described damage‐control role.^[^ ^17^ ^]^ Dissection of the above‐mentioned downregulated processes unveiled a host of proteins (GSTP1, NQO1, PRDX3, PRDX1, ADH5, SRXN1, DHFR, GSTM2, ESD, ALDH1A1, GSTM3, MTARC2, AKR1B10, PARK7) with central roles in cellular protection against various types of harmful metabolites, especially reactive oxygen species (ROS) (Figure S5B, Supporting Information).^[^ ^30^, ^31^, ^32^, ^33^, ^34^, ^35^, ^36^, ^37^, ^38^ ^]^ Amongst these, PRDX3, ALDH1A1, NQO1, and AKR1B10 emerged as the most downregulated proteins in our T98G^Res^ resistant GBM model, upon silencing of PANK4 (Figure 7D). We next validated the results of the TMT‐based proteomic analysis by assessing the expression levels of these proteins. We confirmed their decreased expression following PANK4 depletion, both in T98G^Res^ and U87MG^Res^ cells (Figure 7E). Overexpression of PANK4 in these cells, resulted in increased protein expression of PRDX3, NQO1 and AKR1B10 (Figure S5C, Supporting Information). We next examined the global proteomic changes in PANK4‐depleted cells treated with TMZ (Figure 7F). Enrichment analysis of significantly downregulated proteins, in these cells (Figure 7G), revealed that “cellular response to oxidative stress” was one of the top 20 significantly downregulated biological processes, resulting in deregulation of a subset of proteins, as shown in Figure S5D, Supporting Information. To further support our observations, we also ran Gene Set Enrichment Analysis (GSEA) on all PANK4‐modulated proteins and found a significant downregulation of the GO “cellular response to oxidative stress” process upon PANK4 knockdown, both alone and in combination with TMZ (Figure S5E,F, Supporting Information, respectively). Similar results were also obtained following TMZ treatment (Figure S5G, Supporting Information). Finally, we validated some of the highly downregulated proteins (PRDX3, PDGFD, and ALDH3B1) identified in PANK4‐depleted T98G^Res^ cells treated with TMZ (Figure 7H). Consistent with the TMT data, a decreased protein expression of PRDX3, PDGFD, and ALDH3B1 was observed both in PANK4‐depleted T98G^Res^ and U87MG^Res^ cells treated with TMZ, further corroborating our TMT results (**Figure** **8**I). Collectively, these data reveal a link between PANK4 and a set of proteins of the cellular detoxification system and suggest a possible role for PANK4 in the cellular response to toxic substances and oxidative stress.

Figure 7TMT‐based proteomic analysis reveals reduced cell detoxification response upon PANK4 knockdown. A) Schematic representation of the Tandem Mass Tag (TMT) proteomic experiment. The following four conditions were assessed in T98G^Res^ cells: siCTRL DMSO, siCTRL TMZ, siPANK4 DMSO, and siPANK4 TMZ (n = 3 biological replicates). Figure was created with BioRender.com. B) Volcano plot of differentially expressed proteins following PANK4 knockdown in T98G^Res^ cells (siPANK4 DMSO), highlighting statistically significant changes (*p* ≤ 0.05) in protein abundance compared to control (siCTRL DMSO). The ‐Log_10_(*p*‐values) versus the Log_2_(fold change) in protein abundance are plotted. Horizontal line represents the significant threshold (*p* = 0.05). Red and blue circles indicate significantly up‐ or down‐regulated proteins, respectively. Grey circles indicate proteins with non‐significant changes in abundance following PANK4 silencing; (ns, not significant). Validation of PANK4 protein levels by western blot using tubulin as loading control is shown. C) Dotplot showing the top 20 significantly enriched Gene Ontology (GO) Biological Processes (BP) of downregulated proteins following PANK4 knockdown (siPANK4 DMSO versus siCTRL DMSO). D) Violin plot showing abundance of the 14 significantly downregulated proteins following PANK4 knockdown involved in the GO BP terms: “cellular detoxification”, “cellular response to toxic substance”, and “detoxification”. E) Western blots showing expression of PANK4, PRDX3, ALDH1A1, NQO1, and AKR1B10 in control and PANK4‐depleted T98G^Res^ (left) and U87MG^Res^ cells (right) at 72 and 96 hours. Tubulin was used as loading control. F) Volcano plot of differentially expressed proteins following combined PANK4 knockdown and TMZ treatment of T98G^Res^ cells was generated (siPANK4 TMZ) as in B); statistically significant changes in protein abundance (*p* ≤ 0.05) compared to control (siCTRL DMSO) are shown. G) Dotplot displaying the top 20 significantly enriched GO BP terms of downregulated proteins following combined silencing of PANK4 and treatment with TMZ (siPANK4 TMZ versus siCTRL DMSO). H) Violin plot showing abundance of the 29 significantly downregulated proteins following PANK4 knockdown and TMZ treatment associated with the GO BP term “cellular response to oxidative stress”. I) Western blots showing expression of PANK4, PRDX3, PDGFD, and ALDH3B1 in control and PANK4‐depleted T98G^Res^ (left) and U87MGRes cells (right) treated either with DMSO or TMZ, at 96 hours. Tubulin was used as loading control.

**Figure:**
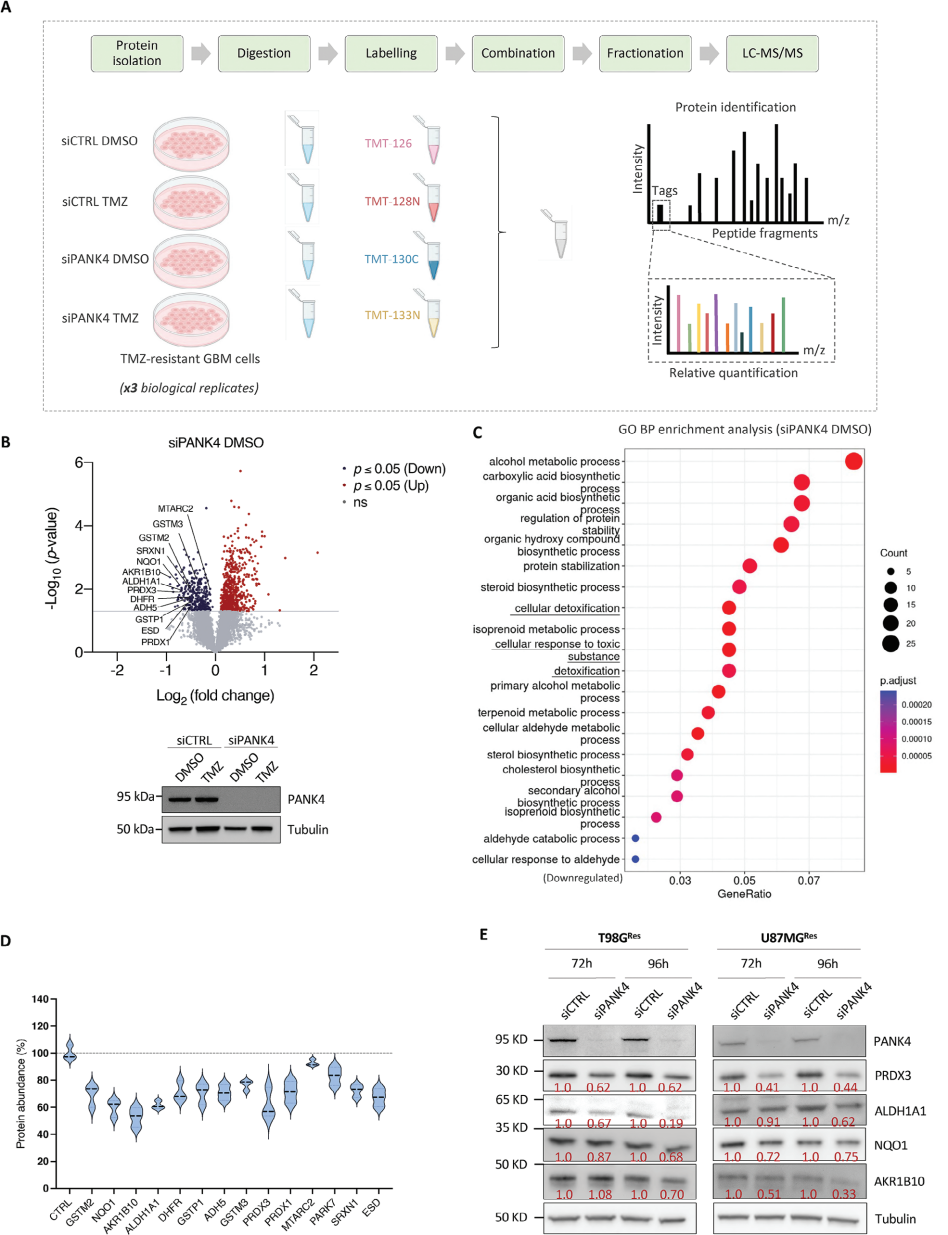

**Figure:**
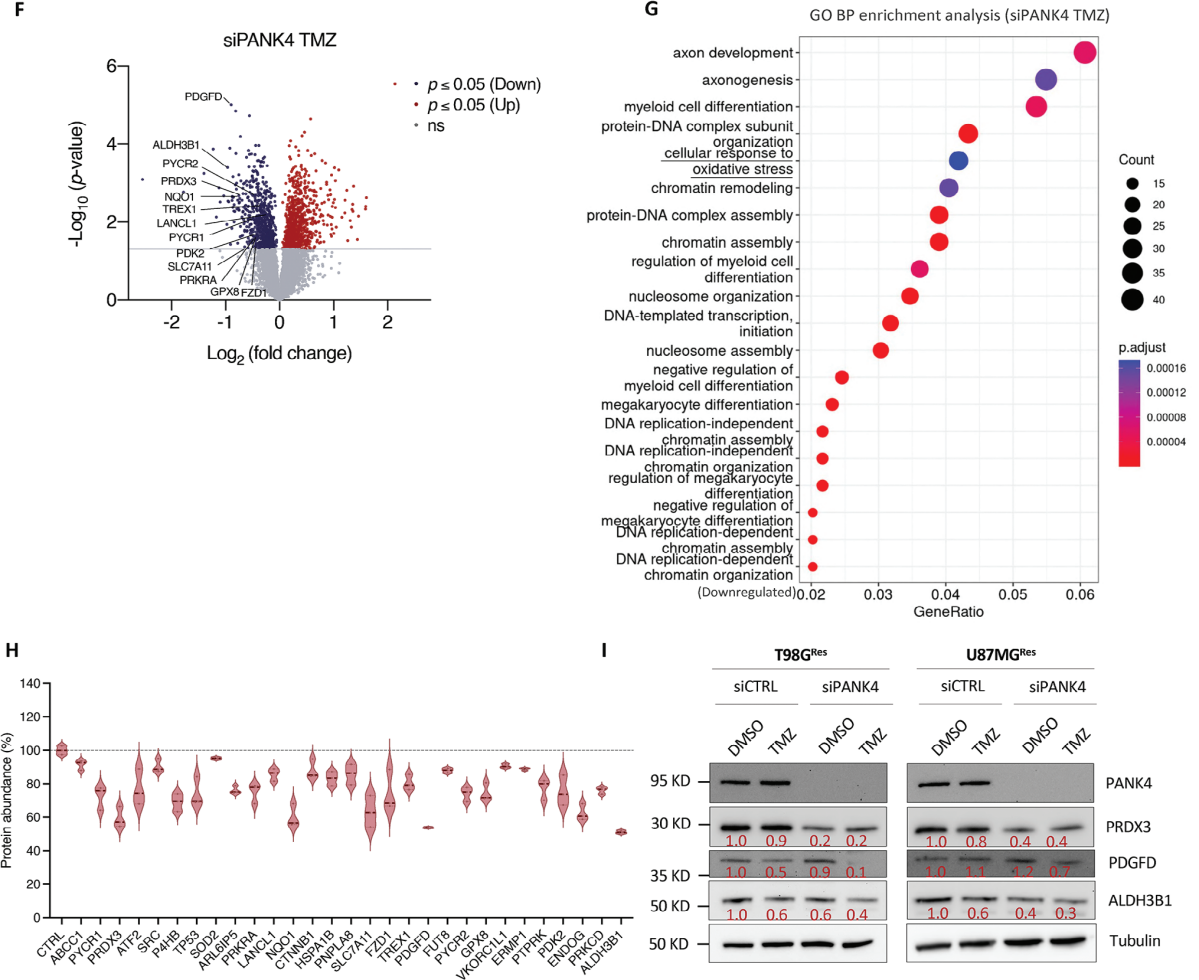

### 2.8 Modulation of PANK4 Affects Intracellular ROS Levels of Resistant GBM Cells

Numerous toxic metabolites, such as ROS, can accumulate within the cell and, if not adequately cleared, these can have detrimental consequences, emphasizing the importance for cells to rely on efficient detoxification systems.^[^ ^39^ ^]^ Most of the downregulated proteins that were identified through our TMT‐based study following PANK4 knockdown (including PARK7, AKR1B10, PRDX1, NQO1, GSTP1, PRDX3, GSTM3, ALDH1A1, SRXN1, and GSTM2), are linked to cellular protection against oxidative stress in GBM,^[^ ^30^, ^31^, ^32^, ^33^, ^34^, ^35^, ^36^ ^]^ and other types of cancers.^[^ ^37^, ^38^, ^40^, ^41^, ^42^ ^]^ To further corroborate PANK4's involvement in the cellular response to oxidative stress, we examined the effects of PANK4 modulation on intracellular ROS levels in our resistant GBM cells. Our results showed that PANK4 silencing resulted in a surge in ROS levels, in both T98G^Res^ and U87MG^Res^ cells (Figure 8A). Conversely, overexpression of PANK4 led to attenuation of intracellular ROS in these cells (Figure 8B), supporting the contribution of PANK4 in modulating oxidative stress. Moreover, the greatest surge in intracellular ROS levels was observed following combined PANK4 silencing and TMZ treatment (Figure 8C). Notably, the accumulation of ROS was markedly reversed in the presence of the ROS scavenger N‐Acetyl‐L‐cysteine (NAC). Next, to examinate whether the increased ROS levels were responsible for the observed effects of PANK4 knockdown and TMZ treatment on cell proliferation, rescue experiments were performed using NAC. Treatment with NAC significantly restored cell proliferation in PANK4‐depleted cells treated with TMZ (Figure 8D,E). These data suggest that PANK4 plays a crucial role in mediating the accumulation of intracellular ROS. Furthermore, treatment with NAC decreased ROS production and reversed the chemo‐sensitizing effect mediated by PANK4 knockdown. Taken together, our data indicate that PANK4 contributes to the cellular detoxification response by modulating a set of proteins with central roles in the antioxidant defense system, by preventing stress overload, including oxidative stress‐induced damage.

### 2.9 The Phosphatase Activity of PANK4 Is Required for TMZ Sensitivity and ROS Accumulation

It has recently been reported that PANK4 is a pseudokinase harboring substitutions of two key residues in the catalytic domain (Glu138Val and Arg207Trp), which are required for its kinase activity.^[^ ^16^ ^]^ Notably, PANK4 is characterized by a DUF89 phosphatase domain that possesses damage‐control functions (**Figure** **9**A).^[^ ^17^ ^]^ By participating to the damage pre‐emption processes (also known as “housecleaning” processes), the DUF89 domain is responsible for the removal of potentially harmful build‐ups of metabolites or side products.^[^ ^17^, ^20^ ^]^ Interestingly, a recent study by Dibble et al. proposed that PANK4's regulation of CoA levels is dependent on its phosphatase activity.^[^ ^18^ ^]^

Figure 9The phosphatase activity of PANK4 is required for TMZ sensitivity and ROS accumulation. A) Schematic diagram showing the PANK4 protein domains. Adapted from Huang et al. (2016).^[^ ^17^ ^]^ B) T98G^Par^ and C) T98G^Res^ cells were transfected with either pCMV6, pCMV6‐PANK4, or the phosphatase‐dead mutant versions of PANK4: pCMV6‐PANK4 (D623A) or pCMV6‐PANK4 (D659A)[18] and treated with sublethal concentrations of DMSO or TMZ after 24 h. Cell proliferation was evaluated at 96 h. Representative images of the proliferation assays are presented alongside. Magnification, 10×. Scale bar, 400 µm. D) T98G^Par^ and T98G^Res^ cells were transfected as in (B) and (C) and treated with sublethal concentrations of DMSO or TMZ after 24 h. ROS levels were evaluated at 96 h. E) Schematic model depicting the role of PANK4 in TMZ resistance in GBM.

**Figure:**
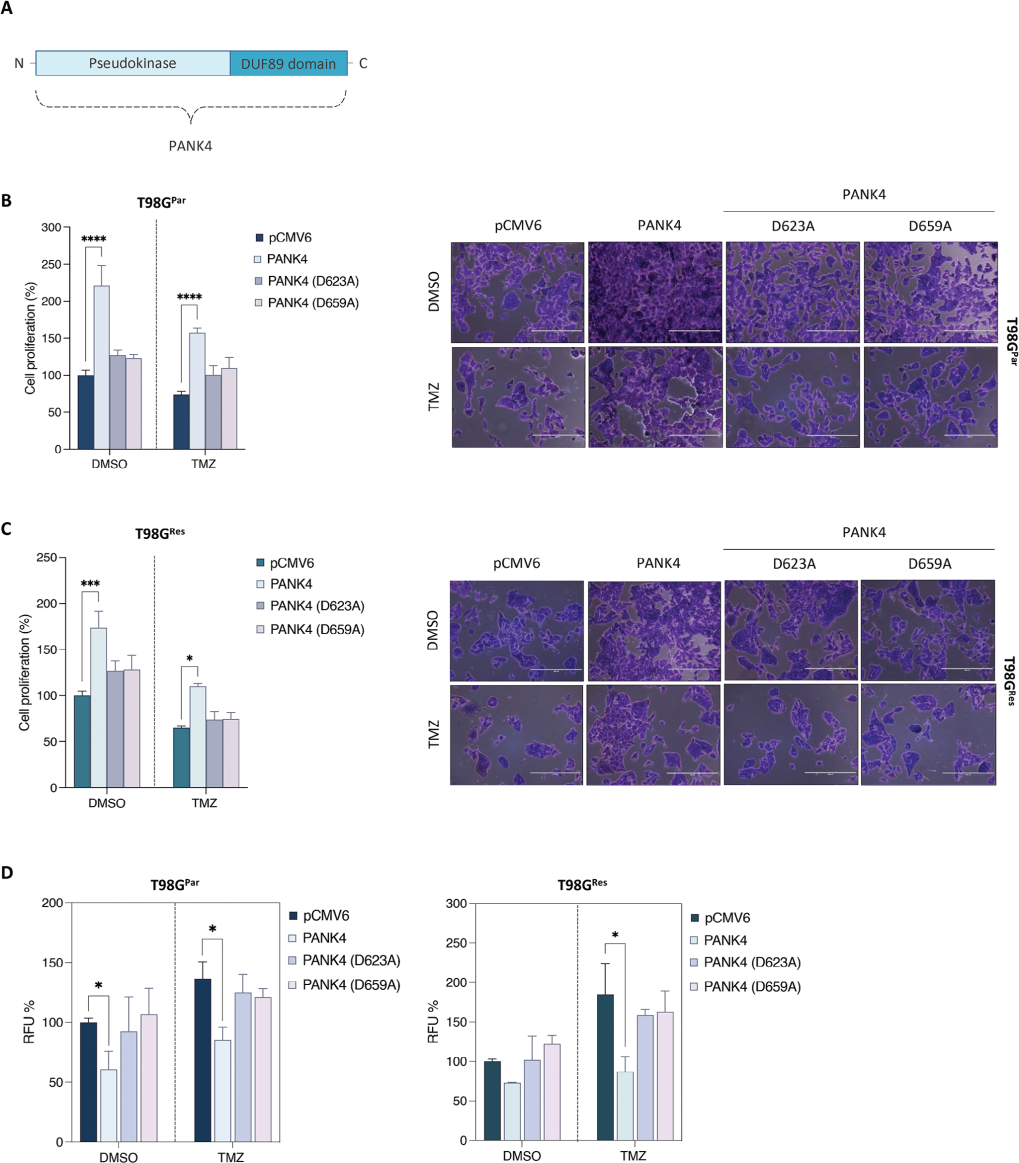

**Figure:**
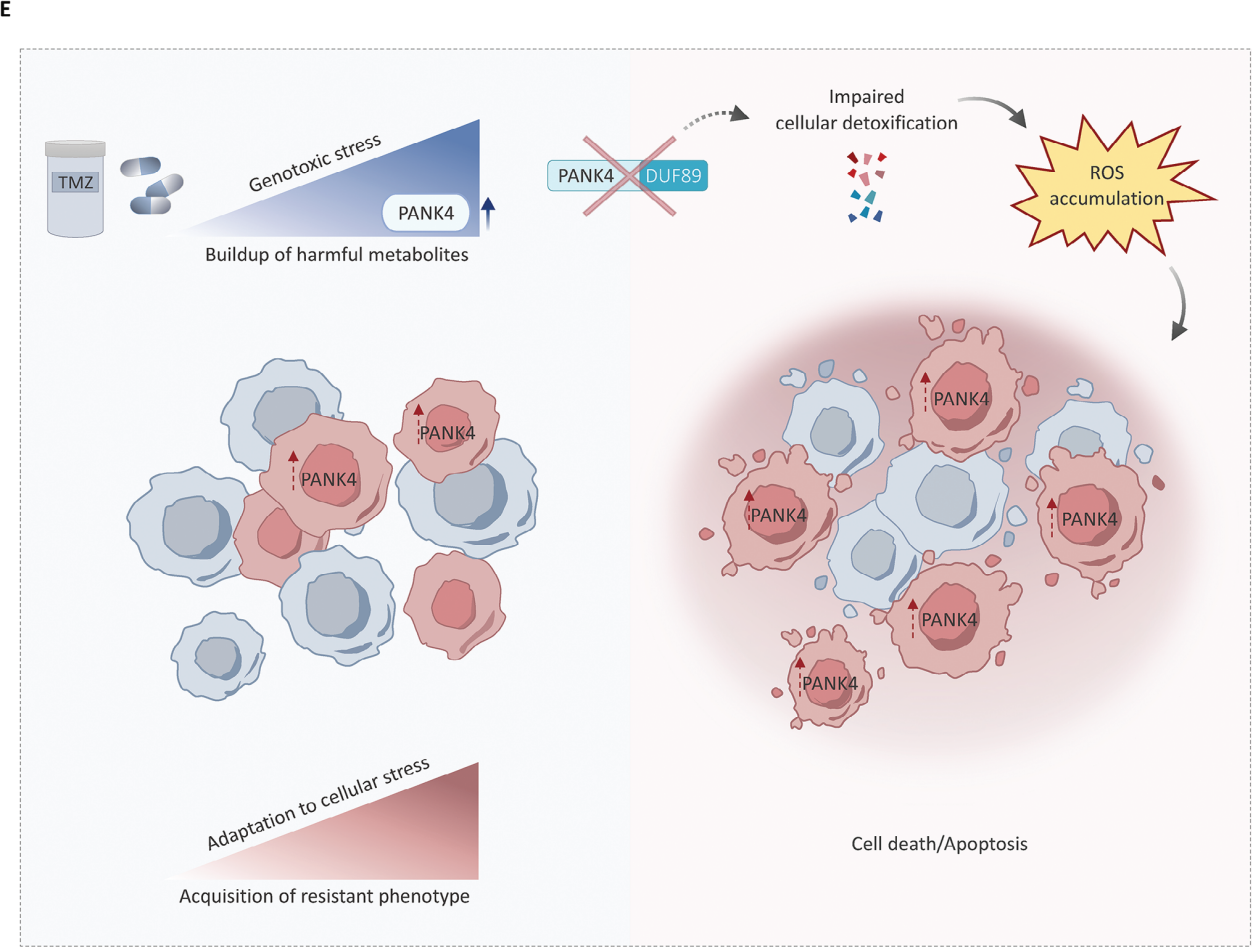

To assess whether the phosphatase activity of PANK4 is required for its ability to modulate sensitivity to TMZ treatment, we overexpressed the wild‐type and two phosphatase‐dead mutants (D623A and D659A) of PANK4 in our TMZ‐resistant GBM cells. Our results showed that PANK4‐mediated increase in cell proliferation is reversed in cells overexpressing PANK4 mutants (D623A and D659A), either alone or in combination with TMZ (Figure 9B,C), suggesting that PANK4 modulates cell sensitivity to the drug in a phosphatase‐dependent manner. In light of PANK4's involvement in the oxidative‐stress response, we next examined whether the effects of PANK4 on ROS production are also dependent on its phosphatase activity. As anticipated, a reduction of ROS levels was observed in cells overexpressing wild‐type PANK4. This effect was abolished after re‐expression of either of the two phosphatase‐dead mutant versions of PANK4 (Figure 9D).

Altogether, these data suggest that PANK4 modulates chemosensitivity of TMZ‐resistant GBM cells to TMZ in a way that is dependent on its phosphatase activity. Furthermore, the phosphatase domain of PANK4 is also essential for the protein's ability to affect intracellular ROS accumulation. Collectively, our data propose a novel protective role for PANK4 in the context of TMZ‐resistant GBM cells. Importantly, loss of PANK4 can tip the balance towards an impaired detoxification response and subsequently lead to ROS accumulation and cell death (Figure 9E).

## 3 Discussion

Resistance to TMZ remains a major challenge in the treatment of GBM, with most patients developing recurrence, and displaying a poor survival rate.^[^ ^1^, ^2^, ^3^ ^]^ While several hallmarks of chemo‐resistance in GBM have been described and intensively studied,^[^ ^43^, ^44^, ^45^, ^46^ ^]^ there are still some poorly explored areas of research, holding great therapeutic potential, that are worthy of investigation. The interest in kinases and pseudokinases has considerably grown over the past years owing to their versatile nature.^[^ ^11^, ^12^, ^13^, ^14^ ^]^ In particular for pseudokinases, despite being regarded as “inert” due to their defective catalytic activity, their active role in physiology and disease, as well as in drug‐resistance,^[^ ^47^, ^48^, ^49^ ^]^ has put them at the center of an ever‐growing and dynamic area of research.

Our study provides evidence supporting a previously unreported role for PANK4 in mediating resistance to TMZ chemotherapy in GBM. Our in vitro data demonstrate that concomitant PANK4 abrogation and TMZ treatment can reverse chemo‐resistance by reducing cell proliferation, colony formation potential and increasing cell apoptosis on a number of TMZ‐resistant GBM cell models, while sensitizing GBM tumors to TMZ treatment in vivo. These results add to our understanding of PANK4 function, an overlooked member of the pantothenate kinase family carrying a kinase domain that has undergone inactivation due to evolutionary mutations.^[^ ^16^, ^17^ ^]^ Because of its lack of catalytic activity, PANK4 has largely been neglected. The limited number of available studies mainly explore its role in the biosynthesis of coenzyme A (CoA) and highlight its potential as a target in pantothenate kinase‐associated neurodegeneration (PKAN) disorders.^[^ ^16^, ^17^, ^18^, ^19^ ^]^ Nevertheless, PANK4 also appears to possess additional roles beyond those already described. Notably, PANK4 is characterized by a DUF89 phosphatase domain that appears to be central to PANK4 function. Intriguingly, the DUF89 domain was reported to confer to the protein its unique features as damage‐control phosphatase, being responsible for clearing the cells from unwanted normal or damaged metabolites that can build up to toxic levels under certain conditions.^[^ ^17^ ^]^ Our data suggest that PANK4 modulates chemosensitivity of TMZ‐resistant GBM cells to TMZ in a phosphatase‐dependent manner. Furthermore, the phosphatase domain of PANK4 is also essential for the protein's ability to affect intracellular ROS accumulation. These results indicate that targeting the phosphatase domain of PANK4 could hold therapeutic potential. However, much remains to be explored in upcoming studies. It is worth mentioning that based on the principle of “guilt by association”,^[^ ^50^ ^]^ the domains of fusion proteins are likely to be functionally related. This is probably why, considering PANKs’ involvement in CoA biosynthesis, studies have mostly focused their efforts to investigate the role of PANK4 in the CoA pathway.^[^ ^16^, ^17^, ^18^, ^19^ ^]^

Our findings suggest that PANK4 could have broader functions by controlling the levels of a wider range of toxic molecules, including but not limited to ROS. To note, a number of metabolites can exert toxic effects and, if not promptly “drained”, can accumulate to toxic levels.^[^ ^39^ ^]^ Our proteomic analysis uncovered a marked downregulation of a host of detoxification proteins in response to PANK4 silencing, such as GSTP1, NQO1, PRDX3, PRDX1, ADH5, SRXN1, DHFR, GSTM2, ESD, ALDH1A1, GSTM3, MTARC2, AKR1B10, PARK7, some of which play crucial roles in cellular protection against oxidative stress in GBM^[^ ^30^, ^31^, ^32^, ^33^, ^34^, ^35^, ^51^ ^]^ and other cancers.^[^ ^37^, ^38^, ^40^, ^41^, ^42^ ^]^ In line with other studies, downregulation of the above‐mentioned proteins has been linked to numerous processes in GBM, such as inhibition of cell proliferation and tumor growth^[^ ^30^, ^31^, ^32^, ^33^, ^34^, ^51^, ^52^, ^53^ ^]^ increased apoptosis^[^ ^31^, ^32^, ^35^, ^51^, ^52^ ^]^ and most importantly, sensitization of GBM cells to treatment with TMZ and/or ionizing radiation.^[^ ^30^, ^33^, ^35^, ^36^, ^53^ ^]^ Similar effects have also been observed in other cancer types.^[^ ^41^, ^54^, ^55^ ^]^

Interestingly, we also showed that PANK4 expression is induced in TMZ‐resistant GBM cells following exposure to the drug, suggesting a potential requirement for PANK4 in the response to TMZ‐induced genotoxic damage. Notably, the DUF89 gene YMR027W in yeast has been reported to be upregulated in response to treatment with the DNA‐damaging agent methyl methanesulfonate.^[^ ^17^, ^56^, ^57^ ^]^ Similarly, its human ortholog, C6orf211 (Armt1), has also been implicated in the response to DNA damage.^[^ ^58^ ^]^

As cancer cells depend on several compensatory mechanisms, especially following potential accumulation of lethal damage, the increase in PANK4 levels after TMZ treatment could provide an advantage to GBM cells. Herein, we propose a mechanism whereas PANK4 depletion compromises the detoxification response in TMZ‐resistant GBM cells, as demonstrated by the downregulation of a number of cellular detoxification proteins. This, alongside the genotoxic stress induced by TMZ leads to a crucial perturbation of the cellular damage response and increased ROS levels, culminating to cell death. Nevertheless, much still needs to be learnt about PANK4, and the extent of its involvement in the cellular detoxification mechanisms at the molecular level has yet to be fully defined. A comprehensive profiling of the implicated toxic metabolites may guide future research efforts and uncover metabolic signatures and vulnerabilities for drug‐resistant GBM cells. Leveraging such vulnerabilities can prove crucial to reverse chemoresistance and restore sensitivity to TMZ, therefore representing a valuable strategy to improve GBM patients’ response to TMZ treatment.

## 4 Conclusion

Our study provides novel insights into chemoresistance in GBM and unveils a protective role for PANK4 in TMZ‐resistant cells. More specifically, in light of the involvement of PANK4 in the detoxification and oxidative stress cellular response, depletion of the protein crucially shifts the balance towards an impaired stress response, exacerbating the damage caused by TMZ and ultimately leading to cell death. In summary, PANK4 represents a vulnerability that could be exploited to restore sensitivity to the drug.

## 5 Experimental Section

### Reagents

TMZ (#T2577‐25MG) was purchased from Sigma–Aldrich and resuspended in DMSO (ThermoFisher Scientific, #D/4125/PB08). PANK4 (#12055, 1:1000), MCL1 (#5453, 1:1000), caspase‐3 (#9665, 1:1000), cleaved caspase‐3 (#9664, 1:1000), GAPDH (#5174, 1:1000), NQO1 (#3187, 1:1000), ALDH1A1 (#36671, 1:1000) as well as antirabbit (#7074P2, 1:4000) and antimouse (#7076P2, 1:4000) HRP‐linked antibodies were purchased from Cell Signaling Technology. PRDX3 (#STJ115041, 1:1000), AKR1B10 (#STJ195042, 1:1000), and Cytochrome c (#STJ97419, 1:1000) were purchased from St. John's Laboratory. PDGFD (#14075‐1‐AP; 1:1000) and ALDH3B1 (#19446‐1‐AP, 1:1000) were purchased from Fisher Scientific. Alpha‐tubulin (#A01410‐100, 1:1000) was purchased from GenScript. For IHC, the anti‐Ki‐67 antibody (#ab15580, 1:150) was purchased from Abcam, the anti‐PANK4 antibody (#HPA027961, 1:300) was purchased from Sigma–Aldrich, total caspase‐3, (#9662, 1:250) and cleaved caspase‐3 (#9661, 1:500) were purchased from Cell Signaling. The pCMV6‐PANK4 overexpressing plasmid (#RC208116) and the pCMV6 empty vector (#PS100001) were purchased from Origene. pCMV6‐PANK4 (D623A) and pCMV6‐PANK4 (D659A) were generated and purchased from Genscript.^[^ ^18^ ^]^ The pLKO.1‐puro PANK4 shRNA (#SH0111; targeting sequence: GGACTCTTCTGCTTGTCACTT) and the pLKO.1‐puro non‐targeting scrambled shRNA (#SHC016) were purchased from Sigma‐Aldrich. The pMD2.G (#12259) and psPAX2 (#12260) packaging plasmids were obtained from Addgene. N‐Acetyl‐L‐cysteine (NAC) was purchased from Sigma–Aldrich (#A9165). All other reagents, if not otherwise specified, were purchased from ThermoFisher Scientific.

### Cell Lines and Culture Conditions

The TMZ‐resistant GBM cell lines (U87MG^Res^, T98G^Res^, and LN229^Res^) and their parental counterparts (U87MG^Par^, T98G^Par^, and LN229^Par^) used in this study were derived from the “Resistant Cancer Cell Line (RCCL) collection” (https://research.kent.ac.uk/industrial‐biotechnology‐centre/the‐resistant‐cancer‐cell‐line‐rccl‐collection/; University of Kent, UK)^[^ ^21^ ^]^ and established by continuous exposure to increasing drug concentrations as described before.^[^ ^59^ ^]^ These cell lines as well as the control and stably PANK4‐depleted T98G^Res^ cells were cultured in Iscove's Modified Dulbecco's Medium (IMDM; ThermoFisher Scientific, #21980‐032) supplemented with 10% fetal bovine serum (FBS; Sigma–Aldrich, #F7524‐500ML), 1% Penicillin‐Streptomycin solution (Sigma–Aldrich, #P0781‐100ML) and 4 mm L‐glutamine (Sigma–Aldrich, #G7513). The U251^Res^ and U251^Par^ cell lines were kindly provided by Dr Corinne Griguer (University of Iowa, Iowa City, USA) and were generated as previously described.^[^ ^22^ ^]^ These cells were grown in DMEM/F‐12 medium (ThermoFisher Scientific, #11320‐033) supplemented with 7% heat‐inactivated FBS (Sigma–Aldrich, #F7524‐500ML) and 1% Penicillin‐Streptomycin solution (Sigma–Aldrich, #P0781‐100ML). All drug‐resistant cells mentioned above were maintained in culture in the presence of TMZ as previously described (https://research.kent.ac.uk/industrial‐biotechnology‐centre/the‐resistant‐cancer‐cell‐line‐rccl‐collection/).^[^ ^21^, ^22^ ^]^ The U3027MG and U3031MG patient‐derived GBM cell lines were obtained from the “Human Glioblastoma Cell Culture (HGCC) biobank” (https://www.hgcc.se; Uppsala University, Uppsala, Sweden).^[^ ^23^ ^]^ These cell lines were cultured in Neurobasal (ThermoFisher Scientific, # 21103‐049) and DMEM/F‐12 GlutaMAX (ThermoFisher Scientific, #31331‐028) medium (1:1), supplemented with B‐27 (ThermoFisher Scientific, #12587010), N2 (ThermoFisher Scientific, #17502048), EGF (PeproTech, #AF‐100‐15‐100UG), FGF (PeproTech, #100‐18B‐100UG), 1% Penicillin‐Streptomycin solution (Sigma–Aldrich, #P0781‐100ML) and grown on laminin‐coated Corning Primaria Cell Culture plates (Corning, #353846 & 353872), as previously described.^[^ ^23^ ^]^ HEK‐293T cells were purchased from ATCC and maintained in Dulbecco's Modified Eagle's Medium (DMEM; Sigma–Aldrich, #D6046‐500ML) supplemented with 10% FBS (Sigma–Aldrich, #F7524‐500ML) and 1% Penicillin–Streptomycin solution (Sigma–Aldrich, #P0781‐100ML). All cell lines were incubated at 37 °C with 5% CO_2_ and regularly subjected to *mycoplasma* testing. Treatments using TMZ were performed at the following concentrations: T98G^Par^ (200 µm), T98G^Res^ (300 µm), U87MG^Res^ (400 µm), and U251^Res^ (150 µm).

### Kinome‐Wide RNAi Screen

The “Silencer Select Human Kinase siRNA Library V4” (ThermoFisher Scientific, #4397918), targeting 709 human kinase and kinase‐related genes was used. U87MG^Res^ cells (3000/well) were reverse transfected in 96‐well plates with either a pool of 3 siRNAs targeting each gene of the library (25 nm/siRNA) or nontargeting negative control siRNAs (ThermoFisher Scientific, #4390844). The Lipofectamine 3000 transfection reagent (ThermoFisher Scientific, #L3000015) was used according to the manufacturer's instructions. Twenty‐four hours after transfection, cells were treated with either DMSO or a sublethal dose of TMZ (IC_20_), and incubated for 96 hours. Cell proliferation was determined using the CyQUANT Direct assay (ThermoFisher Scientific, #C35011), following the manufacturer's instructions. Two independent primary screens (biological repeats) were performed. Data were background corrected and normalized to their respective control (siCTRL DMSO). Normalized values were used to calculate z‐scores as previously described.^[^ ^60^ ^]^ Gene candidates displaying an effect of >20% on cell proliferation alone were excluded from further analysis.

### PANK4 Silencing and Overexpression

Cells were reverse transfected with a pool of 3 siRNAs (25 nm each) using the Lipofectamine 3000 transfection reagent (ThermoFisher Scientific, #L3000015), according to the manufacturer's instructions. Nontargeting negative control siRNA (ThermoFisher Scientific, #4390843) and PANK4 siRNAs (ThermoFisher Scientific, #4392420; IDs: s224353, s30501, s30502) were used. Briefly, a mix of siRNAs, Opti‐MEM medium (ThermoFisher Scientific, #31985062) and Lipofectamine 3000 was prepared following the manufacturer's instructions. After formation of the transfection complexes, the transfection mix was spotted into the wells and cells were subsequently seeded. For PANK4 overexpression, cells were seeded into wells and transfected with the pCMV6‐PANK4 overexpressing plasmid (Origene, #RC208116) or the pCMV6 empty vector (Origene, #PS100001), using the Fugene HD transfection reagent (Promega, #E2311), according to the manufacturer's instructions. To achieve long‐term PANK4 knockdown, lentiviral‐mediated shRNA transfection was performed. Briefly, HEK‐293T cells were transiently co‐transfected with pLKO.1‐puro PANK4 shRNA (targeting sequence: GGACTCTTCTGCTTGTCACTT) (Sigma–Aldrich, #SH0111) or pLKO.1‐puro nontargeting scrambled shRNA (Sigma–Aldrich, #SHC016), and pMD2.G, psPAX2 packaging plasmids (Addgene, #12259 and #12260, respectively). Transient transfection was performed using Lipofectamine 3000 (ThermoFisher Scientific, #L3000015), as described above. Nonreplicating viral particles were harvested and concentrated using PEG‐it (5x) (System Biosciences, #LV810A‐1) overnight at 4 °C. The concentrated virus was dispensed to T98G^Res^ cells, and TransDux (200x) (System Biosciences, #LV850A‐1) was added to increase transduction efficiency. Seventy‐two hours post transduction, cells were incubated in the presence of 0.8 µg mL^−1^ puromycin (Gibco, #A1113803) to ensure effective positive selection. For all the experiments performed, PANK4 silencing and overexpression were confirmed by western blotting, as indicated in the respective figure legends.

### Cell Proliferation Assays

Briefly, cells were reverse transfected with siCTRL or siPANK4, as described above. After 24 hours, cells were treated with DMSO or TMZ, as specified in the figures and their respective legends. Cell proliferation was evaluated by the CyQUANT Direct assay (ThermoFisher Scientific, #C35011), following the manufacturer's instructions. Fluorescence intensity was measured on a SpectraMax i3x microplate reader (Molecular Devices). Alternatively, the crystal violet assay was used. Following fixation of cells with 4% paraformaldehyde solution (Santa Cruz Biotechnology, #sc‐281692) in 1x PBS, and staining with 0.5% crystal violet (ThermoFisher Scientific, #B21932.14), absorbance was measured using the GloMax‐Multi detection system (Promega).

### Clonogenic Survival Assays

Clonogenic survival assays were performed as previously described.^[^ ^61^, ^62^ ^]^ Briefly, cells were transfected with either siCTRL or siPANK4 and subsequently seeded at predetermined densities. After 24 h, cells were treated with DMSO or TMZ, as indicated. When colony size reached more than 50 cells per colony, cells were fixed with 4% paraformaldehyde solution (Santa Cruz Biotechnology, #sc‐281692) in 1× PBS, followed by staining with 0.5% crystal violet (ThermoFisher Scientific, #B21932.14). The surviving fraction was determined using the plating efficiencies of the respective controls as reference.

### Cell Death and Apoptosis

The assay was performed as previously described.^[^ ^63^, ^64^ ^]^ Cells were transfected with either siCTRL or siPANK4 as described above and subjected to drug treatments as specified in the corresponding figures and figure legends. After 96 h, cells were stained using the Muse Annexin V Dead Cell Kit according to the manufacturer's instructions (Luminex, #MCH100105). Cells were then analyzed using the Muse Cell Analyzer (Millipore).

### ROS Detection

The assay was performed using the DCFDA/H2DCFDA – Cellular ROS Assay Kit (Abcam, #ab113851). Briefly, cells were transfected as described above and treated with a sublethal concentration of TMZ as previously determined, where specified. After 72 or 96 hours cells were stained with DCFDA solution according to the manufacturer's instructions and fluorescence was measured using the BMG Labtech CLARIOstar Microplate Reader at Ex/Em = 485/535 nm. Where indicated, the ROS‐Glo H_2_O_2_ kit (Promega, #G8820) was used following the manufacturer's instructions. Relative luminescence units (RLU) were determined using the GloMax Luminometer.

### RNA Extraction and RT‐qPCR

Total RNA was extracted using the PureLink RNA Mini Kit (Invitrogen, #12183018A) following the manufacturer's instructions.^[^ ^60^, ^65^ ^]^ All RNA samples were subjected to DNase treatment. The concentration and purity of RNA was determined using a Nanodrop 2000 spectrophotometer (ThermoFisher Scientific). Complementary DNA (cDNA) synthesis was performed using the High‐Capacity cDNA Reverse Transcription Kit according to the manufacturer's instructions (ThermoFisher Scientific #4368814). Quantitative real‐time PCR was carried out using the SYBR green gene expression assay (Applied Biosystems, #4367659). Samples were run on a StepOne thermal cycler (Applied Biosystems) and analyzed with the SDS 1.9 software (Applied Biosystems) (*n* = 3 biological replicates and *n* = 3 technical replicates). GAPDH was used as an internal control. Primer sequences are listed in Table S4 (Supporting Information).

### Western Blotting and Fractionation Experiment

Western Blotting was performed as previously described.^[^ ^60^, ^63^, ^66^ ^]^ Briefly, cells were lysed in RIPA buffer (Sigma–Aldrich, #R0278) supplemented with protease and phosphatase inhibitors (Roche, #11 697 498 001 and #4 906 845 001, respectively). Protein concentration was determined using the Pierce BCA protein assay kit (ThermoFisher Scientific, #23 227). Proteins were resolved by SDS‐PAGE and transferred onto a nitrocellulose transfer membrane (ThermoFisher Scientific, #IB23001) using the iBlot 2 dry blotting system (ThermoFisher Scientific, #IB21001). Following blocking of membranes in TBS containing 0.1% (v/v) Tween 20 and 5% (w/v) nonfat milk for 1 h, incubation with primary antibodies was performed overnight at 4 °C. Antimouse (#7076P2, 1:4000) and antirabbit (#7074P2, 1:4000) horseradish peroxidase (HRP)‐conjugated secondary antibodies were used (Cell Signaling Technology) and binding was detected using the SuperSignal West Pico PLUS chemiluminescent substrate (ThermoFisher Scientific, #34577). Emission was captured using the UVP ChemStudio Imaging Systems (Analityk jena). Densitometric analysis of western blots was performed using the ImageJ software.

The fractionation experiment for separation of mitochondrial and cytosolic fractions was performed using the Mitochondria Isolation Kit for Cultured Cells (Thermofisher, # 89874) according to the manufacturer's instructions. Isolated mitochondria were then lysed in RIPA buffer as described above for downstream application (western blotting).

### Animal Experiments

NOD.Cg‐Prkdcscid Il2rgtm1Wjl/SzJ (stock no: 005557; NSG)^[^ ^67^, ^68^ ^]^ mice were purchased from the Jax repository (Bar Harbor, ME, USA) and bred in‐house in individually ventilated cages under specific pathogen‐free conditions. All animal studies were performed in full compliance with FELASA (Federation of Laboratory Animal Science Associations) recommendations in the Animal House Facility of the Biomedical Research Foundation of the Academy of Athens (BRFAA, Greece). All procedures for the care and treatment of the animals were approved by the Institutional Committee on Ethics of Animal Experiments. The license for the animal handling protocol for this project is: 1385947/27‐12‐2022. To produce the ectopic tumor xenograft model, 10 × 106 T98GRes cells in 10% matrigel (Corning) were subcutaneously injected in the right and left flank of mice. After establishing palpable tumors (≈30 mm^3^), mice were randomly assigned to groups. Tumor volume was measured twice a week with caliper and calculated as V = a × b2/2, “a” being the largest diameter, “b” the smallest. Tumor specimens up to 50 mm^3^ from nontreated mice were transplanted subcutaneously in new NSG mice under anesthesia in order to produce mirror images of the primary tumor.

### In Vivo Transfections

In Vivo Ready nontargeting negative control siRNA (ThermoFisher Scientific, #4404020) and PANK4 siRNAs (ThermoFisher Scientific, #4404010; HPLC‐IVR IDs: s224353, s30501) were used in this study. siRNAs were encapsulated using Invivofectamine 3.0 reagent (ThermoFisher Scientific, #IVF3001), a cationic liposome‐based formulation, according to the manufacturer's instructions. Animals were anesthetized using an intraperitoneal injection of ketamine (100 mg kg^−1^) and xylazine (12 mg kg^−1^). The injection site was swabbed with 70% ethanol prior to injection. siRNA:Invivofectamine 3.0 complexes were injected intratumorally at a concentration of 6 µg per tumor at day 1 and 5. The siRNA concentrations used were based on previously published studies^[^ ^69^, ^70^ ^]^ and knockdown efficiency was assessed by western blot.

### In Vivo TMZ Treatments

TMZ (MedChemExpress, #HY‐17364/CS‐0943) was administered intraperitoneally (IP) at a concentration of 1.5 mg kg^−1^ on day 2 and every other day for the following 12 days. The concentration of TMZ used was based on the dose response curve generated after IP administration of TMZ at concentrations of 0 (vehicle), 1, 3, and 10 mg kg*^−^* ^1^. TMZ was dissolved in 5% DMSO/5% solutol (Sigma–Aldrich) in PBS. At the end of each treatment, mice were euthanized in accordance with standard protocols. A small part of the freshly dissected tumor was fresh frozen for molecular analysis and the rest was fixed in a 10% formalin solution.

### Immunoblotting of Xenograft Tumor Tissues

Tumors were lysed in 8 m UREA per 50 mm TEAB with protease inhibitors (Calbiochem) using mild sonication on ice followed by homogenization with a 26 G syringe. Total protein concentration was determined with the Bio‐Rad protein assay. Lysates were subjected to SDS–PAGE followed by immunoblot analysis. The primary antibodies used were PANK4 (Cell Signaling Technology, #12055, 1:1000) and GAPDH (Cell Signaling Technology, #5174, 1:1000). The antirabbit HRP‐conjugated secondary antibody (Cell Signaling Technology) was used at a 1:4000 dilution. Densitometric analysis of western blots was performed using the ImageJ software.

### Immunohistochemistry of Xenograft Tumor Tissues

Immunohistochemistry (IHC) was performed according to standard procedures.^[^ ^63^, ^71^ ^]^ Rabbit anti‐Ki‐67 antibody (Abcam, #ab15580, 1:150) was used for overnight incubation at 4 °C in humidified chambers. The antirabbit secondary antibody (Cell Signaling Technology, 1:400) was HRP‐conjugated and was detected with DAB (Vector Laboratories).

### Immunohistochemical Analysis of Clinical Specimens

Immunohistochemistry staining for PANK4 and hematoxylin and eosin (H&E) staining were performed as previously described.^[^ ^63^, ^72^, ^73^ ^]^ The anti‐PANK4 antibody (Sigma–Aldrich, #HPA027961) was used at a 1:300 dilution. The study was approved by the ethics committee of Renmin Hospital of Wuhan University (March 15th, 2022). The approval number is: WDRY2022‐K064. Clinical GBM specimens (*n* = 79 GBM, IDH‐wildtype patients) were collected in the cancer center at Renmin Hospital of Wuhan University, and processed at the hospital research laboratories after deidentification of the samples. PANK4 immunoreactivity was assessed semi‐quantitatively on a 0–2 scale, with 0 = negative, 1+ = mild, 2+ = moderate staining (https://www.proteinatlas.org). Percentages of 0, 1+, 2+ cells were recorded. H‐scores were calculated as follows: % of (1+) cells + 2× [% of (2+) cells]. All cases were scored without knowledge of the clinicopathological data. Patients’ information is provided in Table S5 (Supporting Information).

### Sample Preparation for the TMT‐Based Proteomic Experiment

Briefly, T98G^Res^ cells were reverse transfected with either siCTRL or siPANK4. After 24 h, cells were treated with either DMSO or TMZ. Following 96 h, cells were washed (×3 in PBS) and pelleted. Cell pellets were lysed separately with freshly prepared lysis buffer containing 50 mm HEPES, pH 8.0, 2% SDS, 1 mm PMSF, supplemented with phosphatase and protease inhibitor cocktail (Sigma–Aldrich). Samples were thawed at room temperature (RT) for 20 min before heating to 99 °C for 5 min. After cooling to RT, DNA was sheared by sonication. Cell debris was removed by centrifugation at 20000×g for 15 min at 20 °C. Protein concentration was determined using the BCA protein assay kit (Applichem GmbH, Darmstadt, Germany).

### Protein Digestion and Offline Fractionation

FASP digestion was performed according to the procedure described by Wisniewski et al.^[^ ^74^ ^]^ TMT labelling was performed with TMTpro 16‐plex reagents (Lot#WA314599) according to the manufacturer's instructions (Pierce, Rockford, IL, USA). Offline Fractionation of peptides into 12 fractions was performed via RP‐HPLC at high pH as described by Gilar et al.^[^ ^75^ ^]^ After solvent removal in a vacuum concentrator, samples were reconstituted in 0.1% TFA for LC‐MS/MS analysis.

### Liquid Chromatography–Mass Spectrometry Analysis

Mass spectrometry was performed on an Orbitrap Fusion Lumos mass spectrometer (ThermoFisher Scientific, San Jose, CA, USA) coupled to a Dionex Ultimate 3000RSLC nano system (ThermoFisher Scientific, San Jose, CA, USA) via a nanoflex source. Tryptic peptides were separated on a 50 cm, 75 µm i.d. analytical column (self‐packed with ReproSil‐Pur 120 C18‐AQ, 3 µm, Dr. Maisch, Ammerbuch Entringen, Germany) and a 90 min acetonitrile gradient (5–90%) at a flow rate of 230 nL min^−1^. Analysis was performed in a data‐dependent acquisition mode using a TopN dependent scan method with a cycle time of up to 20 scans for precursor ion selection. MS1 data were acquired in the orbitrap at a resolution of 120000 (at 200 m z^−1^). Automatic gain control (AGC) was set to a target of 2.5E4 and a maximum injection time of 86 ms. MS2 spectra were acquired in the orbitrap (FT) using a quadrupole isolation window of 0.5 Da and higher‐energy collision induced dissociation (HCD) at a normalized collision energy (NCE) of 34%. The resolution was 50000 (at 200 m z^−1^) with a fixed first mass of 110 m z, an AGC target of 5E4, and a maximum injection time of 110 ms. Dynamic exclusion for selected ions was 90 s. A single lock mass at m z^−1^ 445.120024 was employed.^[^ ^76^ ^]^

### Proteomic Data Analysis

Protein identification and comparative quantification of TMTpro 16‐plex labelled proteins from MS and MS/MS raw data were performed using the MaxQuant software suit (version 1.6.12.0) (Max Planck Institute of Biochemistry, Planegg, Germany) with the implemented peptide search engine Andromeda^[^ ^77^ ^]^ against a reference proteome database of Homo sapiens (Human/Uniprot proteome ID: UP000005640, Version 7 March 2021). Statistical analysis was performed using the Perseus software (version 1.6.14.0). Unpaired *t*‐test was employed to determine the significance of the observed differences. Differences were considered statistically significant at *p* < 0.05 (95% confidence interval, *p < 0.05; **p < 0.01; ***p < 0.001; ****p < 0.0001). All relevant data are available from the authors upon request. The TMT‐based proteomics data have been deposited to the ProteomeXchange Consortium (http://www.proteomexchange.org)^[^ ^80^ ^]^ via the PRIDE^[^ ^81^ ^]^ partner repository with the dataset identifier PXD040871 and 10.6019/PXD040871.

### Gene Set Enrichment Analysis

Functional annotation and Gene Set Enrichment Analysis (GSEA) were performed using clusterProfiler (v 4.4.4).^[^ ^78^ ^]^ Enrichment representations were plotted with the dotplot, cnetplot, and gseaplot2 functions. Gene Ontology (GO) terms were considered significantly enriched in over‐representation analysis when Benjamini‐Hochberg adjusted *p* values were below < 0.05(*). For all analyses and plots, R (v 4.2.0) and Bioconductor (v 3.15) were used.

### Statistical Analysis

Graphics and statistical analysis were performed using the GraphPad Prism 9 software. Each experiment was conducted at least three times and results are expressed as mean ± SEM, unless otherwise specified. Statistical significance was evaluated using unpaired Student's *t*‐test when two groups were compared. More than two groups were compared using two‐way ANOVA, unless otherwise specified. For assessment of biological reproducibility of the two primary screens, correlation was determined using the Pearson correlation coefficient. The Cooperativity Index (CI) was calculated as previously described.^[^ ^79^ ^]^ CI is equal to the sum of cell death percentages obtained for each single agent, divided by the percentage of cell death upon combined treatment. CI values < 1 indicate a synergistic effect, when CI values = 1 the effect is additive, and CI values > 1 indicate an antagonistic effect. The statistical significance of the Kaplan–Meier survival plots was evaluated by log‐rank analysis. The statistical details and *p*‐values of each experiment are indicated in the corresponding figures and figure legends [p‐values: *p < 0.05, **p < 0.01, ***p < 0.001, ****p < 0.0001 and “ns” indicates not significant (*p* > 0.05)].

## Conflict of Interest

Georgios Giamas is the Founder/Chief Scientific Officer of StingrayBio and an Associate Editor in Oncogene. Justin Stebbing conflicts are listed at https://www.nature.com/onc/editors. The remaining authors declare that they have no conflict of interest.

## Author Contributions

V.V., A.D., A.C., T.G., C.B., and C.K. contributed to methodology; V.V., A.C., M.E., S.K.W., E.K., C.K., F.M.G.P., L.P., and A.K. performed data analysis; L.P., B.X., and Y.Z. helped with collection and IHC staining of clinical specimens; G.L. performed IHC analysis and scoring; V.V., J.S., and G.G. wrote the original draft and edited the final manuscript: G.G. did supervision and acquired funding. All authors have read and agreed to the published version of the manuscript.

## Supporting information

Supporting Information

Supplemental Tables

## Data Availability

The data that support the findings of this study are available in the supplementary material of this article.
