# Supplementary material for: Kinome‐Wide Synthetic Lethal Screen Identifies PANK4 as a Modulator of Temozolomide Resistance in Glioblastoma
Source: Adv Sci (Weinh). 2024 Feb 14;11(15):2306027. doi: 10.1002/advs.202306027 (PMC11022721; doi:10.1002/advs.202306027)
Supplement: Supplementary file 1 — Supporting Information [file ADVS-11-2306027-s002.pdf]

ADVANCED SCIENCE
Open Access

Supporting Information

for *Adv. Sci.*, DOI 10.1002/advs.202306027

Kinome-Wide Synthetic Lethal Screen Identifies PANK4 as a Modulator of Temozolomide Resistance in Glioblastoma

Viviana Vella, Angeliki Ditsiou, Anna Chalari, Murat Eravci, Sarah K. Wooller, Teresa Gagliano, Cecilia Bani, Emanuela Kerschbamer, Christos Karakostas, Bin Xu, Yongchang Zhang, Frances M.G. Pearl, Gianluca Lopez, Ling Peng, Justin Stebbing, Apostolos Klinakis and Georgios Giamas**

Kinome-Wide Synthetic Lethal Screen Identifies PANK4 as a Modulator of Temozolomide Resistance in Glioblastoma

Viviana Vella, Angeliki Ditsiou, Anna Chalari, Murat Eravci, Sarah K. Wooller, Teresa Gagliano, Cecilia Bani, Emanuela Kerschbamer, Christos Karakostas, Bin Xu, Yongchang Zhang, Frances M.G. Pearl, Gianluca Lopez, Ling Peng, Justin Stebbing, Apostolos Klinakis, Georgios Giamas*.*

V. Vella, A. Ditsiou, M. Eravci, C. Bani, G. Giamas

Department of Biochemistry and Biomedicine, School of Life Sciences

University of Sussex

Falmer, Brighton BN1 9QG, UK.

E-mail: vv62@sussex.ac.uk; g.giamas@sussex.ac.uk

A. Chalari, C. Karakostas, A. Klinakis

Center of Basic Research

Biomedical Research Foundation of the Academy of Athens

Athens 11527, Greece

S.K. Wooller, F.M.G. Pearl

Bioinformatics Group, School of Life Sciences

University of Sussex

Falmer, Brighton BN1 9QG, UK

T. Gagliano, E. Kerschbamer

Department of Medicine

University of Udine

Udine 33100, Italy

B. Xu

Cancer Center

Renmin Hospital of Wuhan University

Wuhan, Hubei, 430064, China

Y. Zhang

Department of Medical Oncology, Lung Cancer and Gastrointestinal Unit

Hunan Cancer Hospital/The Affiliated Cancer Hospital of Xiangya School of Medicine, Central South University

Changsha, Hunan, 430064, China

G. Lopez

Division of Pathology

Fondazione IRCCS Ca' Granda - Ospedale Maggiore Policlinico

Milan 20122, Italy

G. Lopez

Department of Biomedical, Surgical and Dental Sciences, University of Milan

Milan 20122, Italy

L. Peng

Department of Respiratory Disease

Zhejiang Provincial People's Hospital

Hangzhou, Zhejiang 310003, China

J. Stebbing

Department of Life Sciences

Anglia Ruskin University

East Road, Cambridge, CB1 1PT, UK

[*]To whom correspondence should be addressed:

Dr Viviana Vella (vv62@sussex.ac.uk) and Prof Georgios Giamas (g.giamas@sussex.ac.uk)

The PDF file includes:

Supplementary Figures 1-5

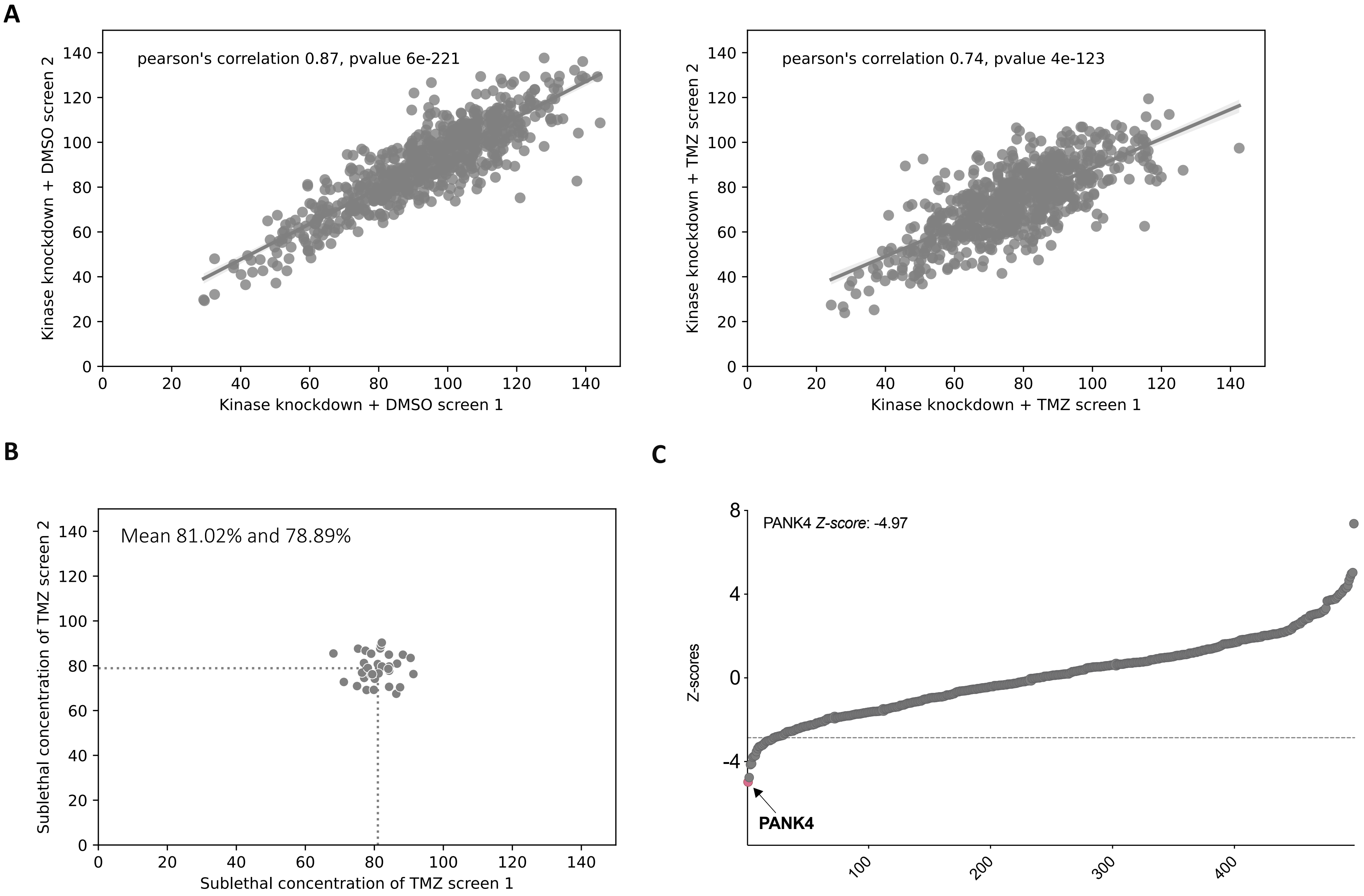

D

	gene	Z scores		gene	Z scores
1	PANK4	-4.9707021	12	IP6K2	-3.2363844
2	MAPK14	-4.758601	13	TSSK6	-3.2174583
3	CLK3	-4.1466784	14	ADCK4	-3.1493006
4	DYRK3	-4.0996745	15	ADPGK	-3.0917456
5	DCLK3	-3.7816765	16	ITPKC	-3.0238886
6	CDC42BPA	-3.7368972	17	TSSK1B	-3.0085243
7	LIMK1	-3.7122275	18	ACVRL1	-2.9963501
8	PLK2	-3.5145072	19	PIK3C2G	-2.9879063
9	ASB10	-3.3787304	20	FRK	-2.9565625
10	ETNK2	-3.2856451	21	RPS6KC1	-2.9315561
11	OXSR1	-3.2743772	22	MST4	-2.8590043

Figure S1. Kinome-wide RNAi screens: correlation and data analysis (**A**) Two independent primary screens were performed. The effect of each gene knockdown following treatment with DMSO (*left*) or TMZ (*right*) on cell proliferation is plotted. Correlation between the two experiments was determined using the Pearson correlation coefficient, showing high degree of correlation (0.87 and 0.74, respectively). (**B**) The effect of the sublethal concentration of TMZ (IC_{20}) on cell proliferation was evaluated for each of the two screens. For the first screen, mean (μ)= 81.02%, standard deviation (σ)= 5.19%. For the second screen, μ= 78.89%, σ = 6.42%. (**C**) Z-scores of synthetic lethal candidate genes generated from the RNAi primary screens with PANK4 being the top-ranking gene; PANK4 *Z-score*: -4.97. (**D**) List of the 22 statistically significant top-ranking genes and their respective Z-scores.

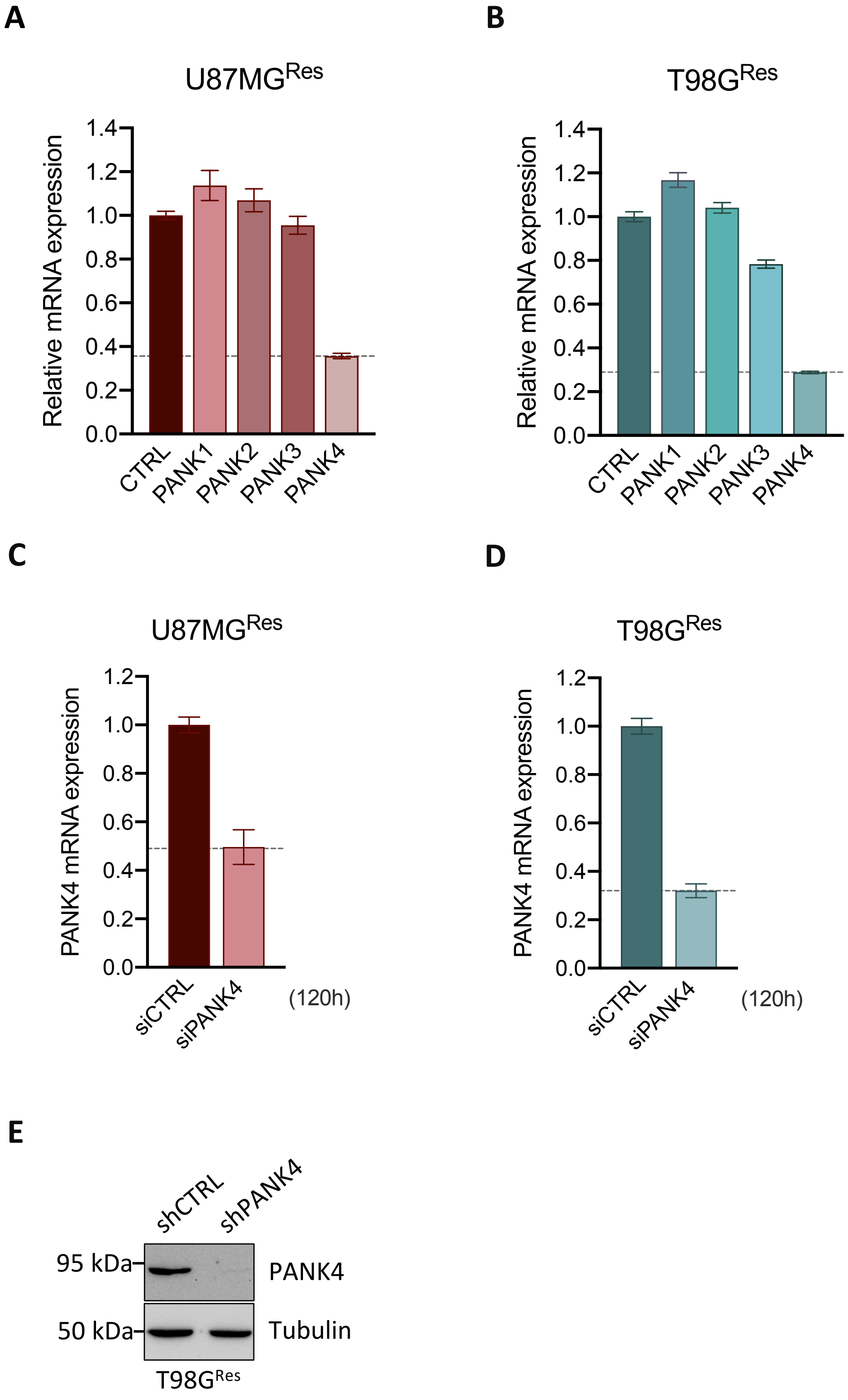

Figure S2. Validation of PANK4 knockdown by RNA-interference. The specificity of the siRNAs used in this study was validated against PANK1, PANK2, PANK3 and PANK4 in (**A**) U87MG^{Res} and (**B**) T98G^{Res} cells following transient transfection with either siCTRL or siPANK4 (24h). (**C** and **D**) PANK4 knockdown efficiency was assessed at 120h (*day 6*) confirming sustained siRNA-mediated gene silencing. GAPDH was used as an internal control (**A**, **B**, **C**, and **D**). (**E**) Western blot showing effective PANK4 knockdown in control and stably PANK4-depleted T98G^{Res} cells (shCTRL/shPANK4). Tubulin was used as loading control.

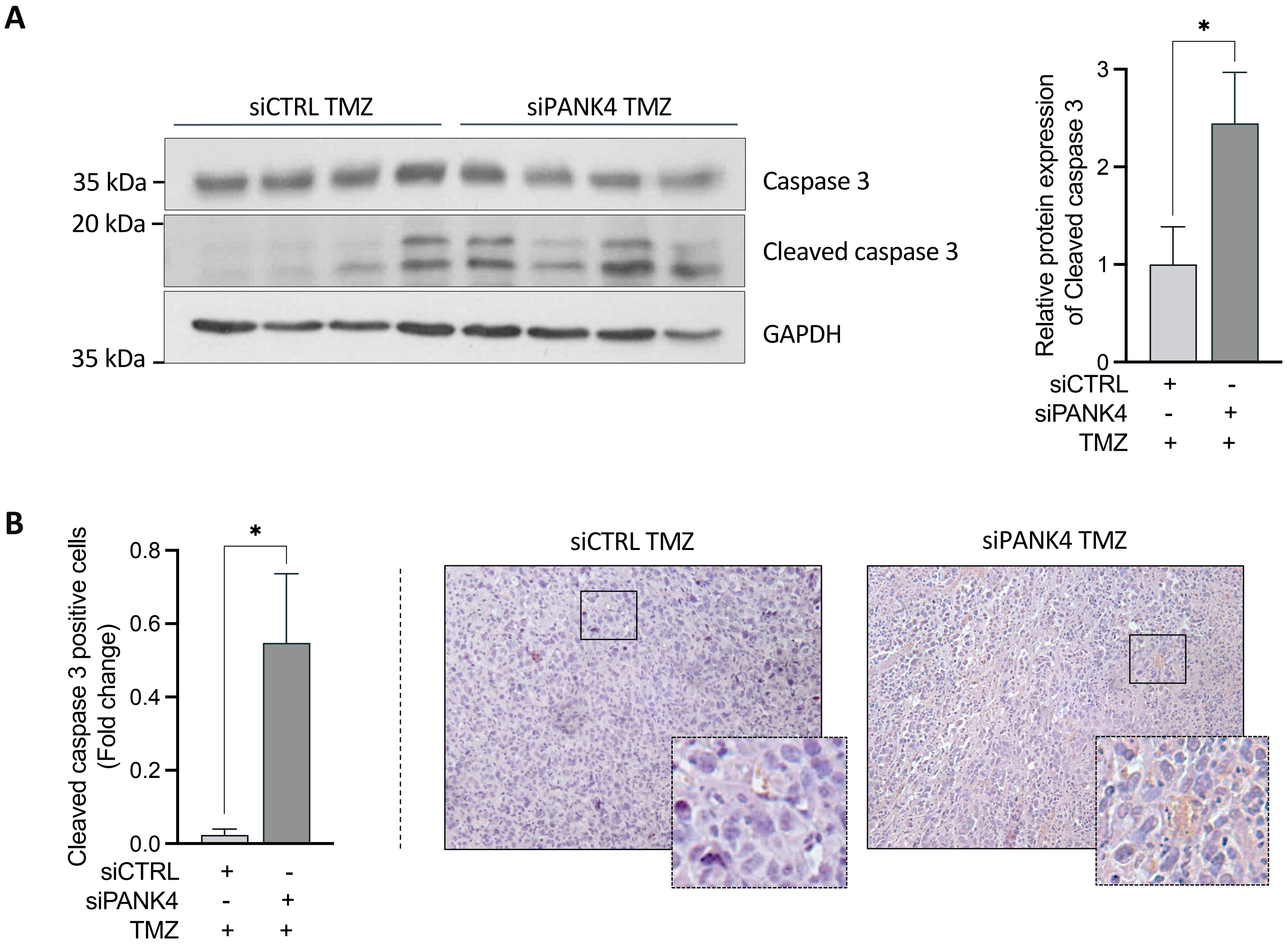

Figure S3. In vivo caspase 3 activation. (**A**) Western blot and densitometric analysis of caspase 3 and cleaved caspase 3 expression in tumour lysates from four distinct tumours for the indicated conditions is shown. GAPDH was used as loading control. (**B**) Immunohistochemical (IHC) evaluation of cleaved caspase 3 expression in tumour sections from T98G^{Res} xenograft mice following treatment with TMZ, with or without PANK4 knockdown. Data represent average of four independent samples per cohort, in duplicate. Representative images of cleaved caspase 3 immunohistochemical staining in harvested tumours from each cohort are presented. Original magnification, x20. Scale bar, 50 μm. Results are expressed as mean ± SEM. Significance was calculated using unpaired Student's t-test; asterisks (*) designate significant differences between conditions indicated with brackets (ns, not significant; *p < 0.05, **p < 0.01).

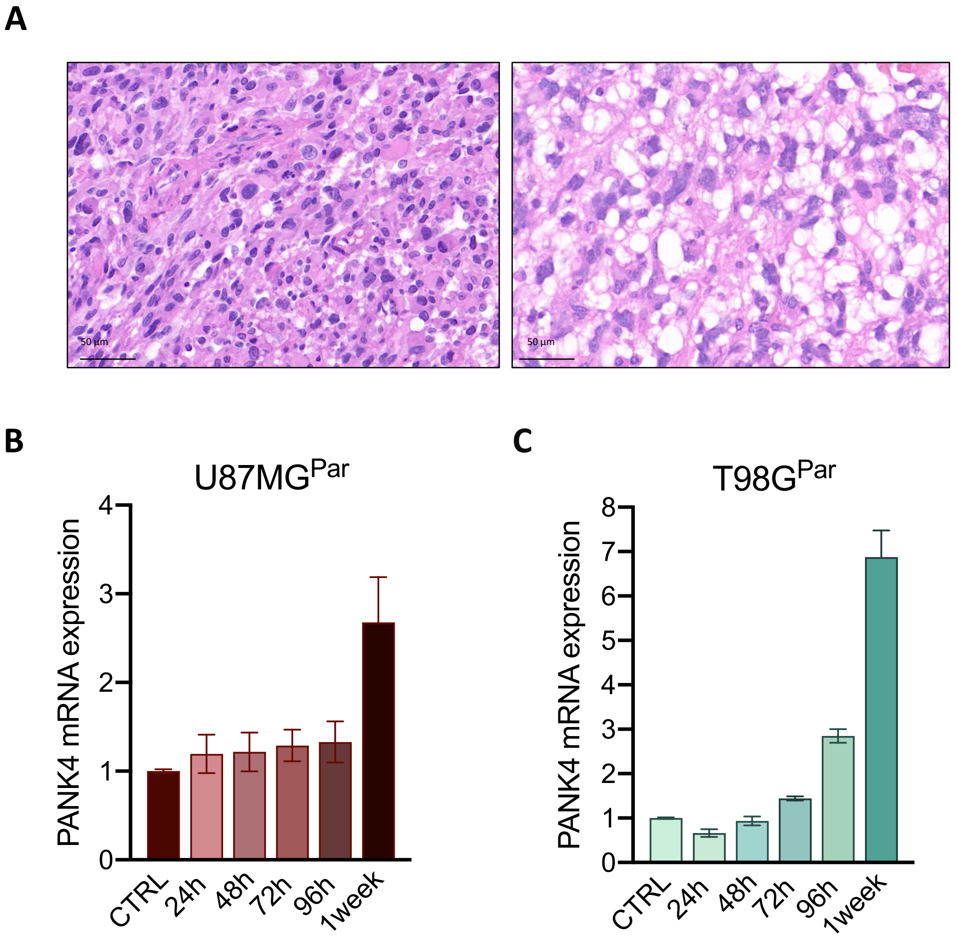

Figure S4. PANK4 expression profile in GBM patient cohorts and its association with TMZ resistance. (**A**) Representative microphotographs of hematoxylin and eosin (H&E) stained tissue sections of two cases of glioblastoma, IDH-wildtype, on which the immunohistochemical analysis (IHC) for PANK4 expression was performed. Scale bar, 50 μm. (**B**) Relative PANK4 mRNA expression in U87MG^{Par} and (**C**) T98G^{Par} cells following treatment with the TMZ concentrations used to generate their resistant counterparts, for the indicated time points. GAPDH was used as an internal control.

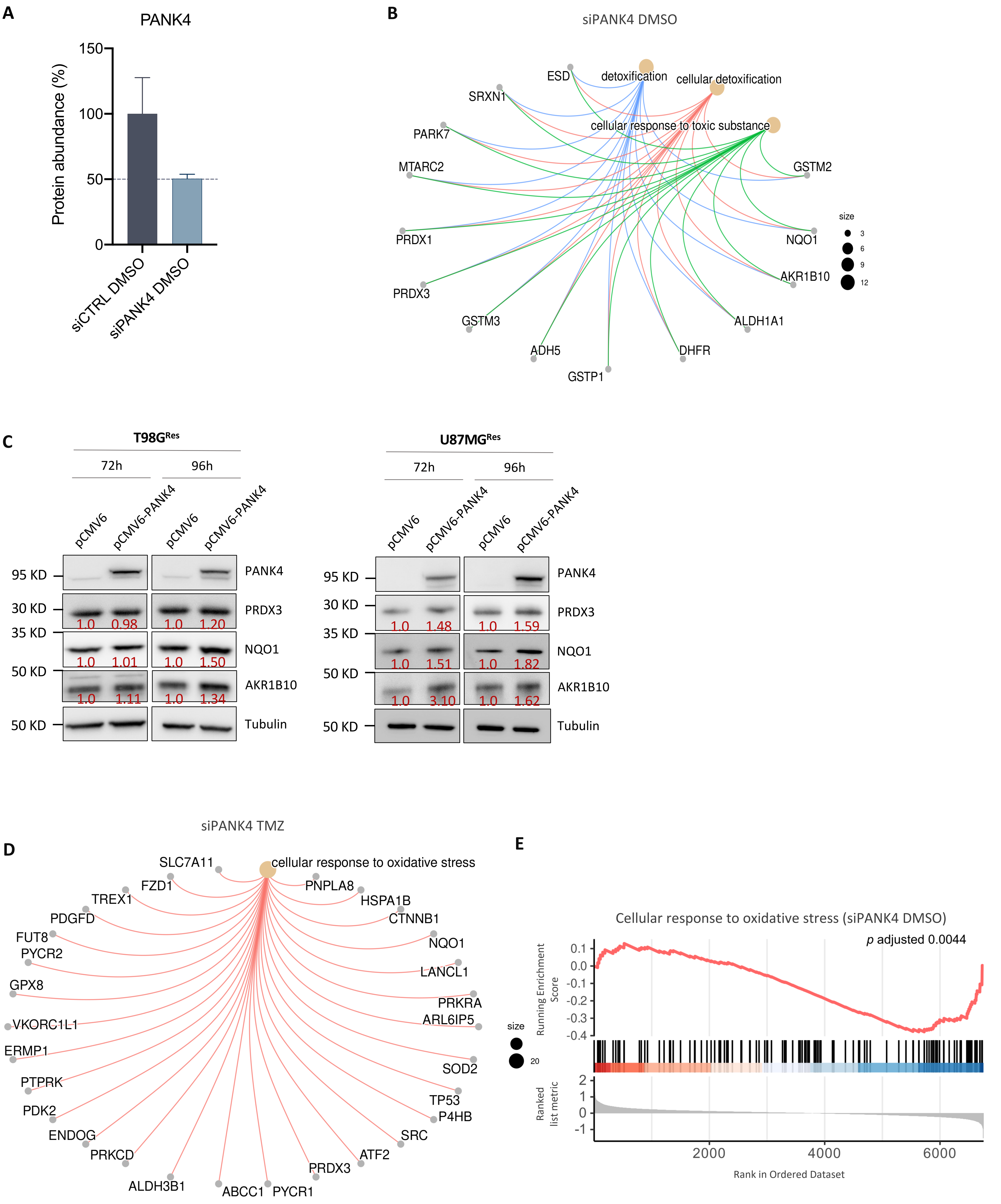

Figure S5. PANK4 depletion affects cellular stress response.

F

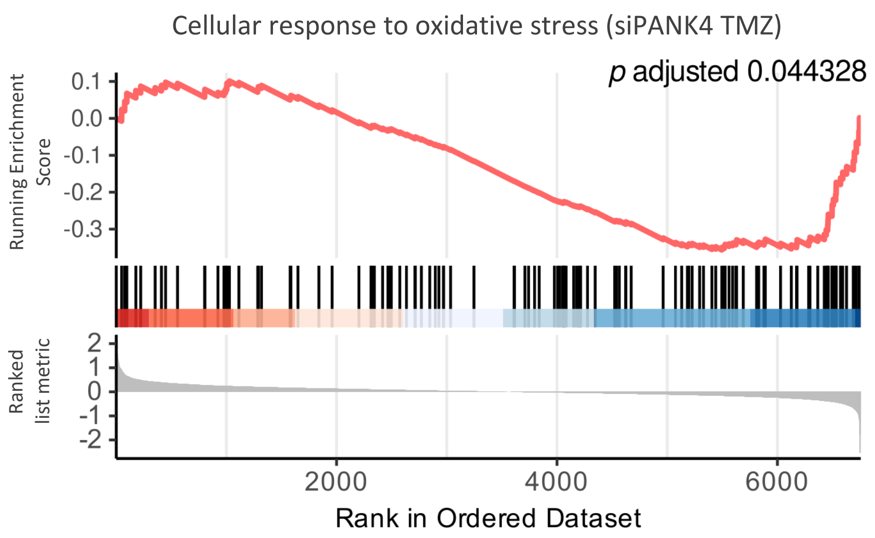

G

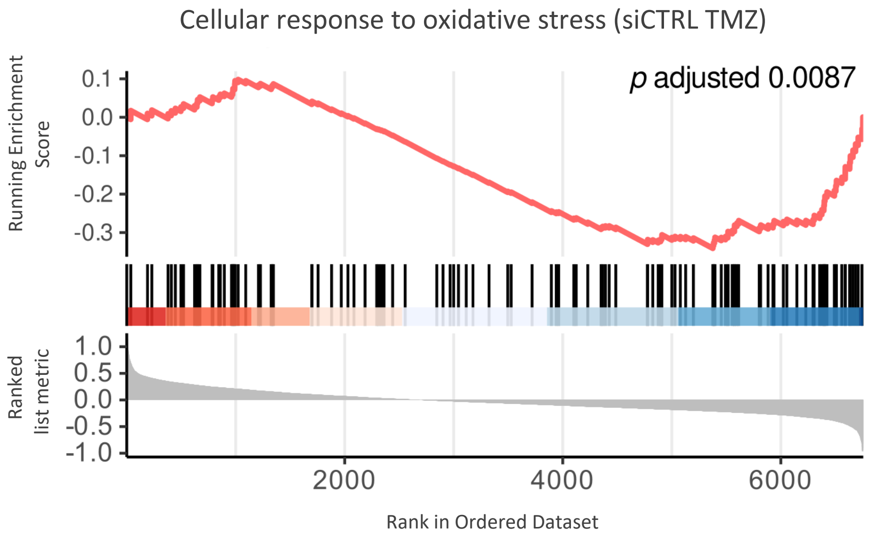

Figure S5. PANK4 depletion affects cellular stress response. (**A**) Percentage of PANK4 protein abundance confirming effective PANK4 protein knockdown in TMT experiment. (**B**) Cnetplot depicting the 14 significantly downregulated proteins involved in the GO BP terms of interest, including “cellular detoxification”, “cellular response to toxic substance” and “detoxification”. (**C**) Western blots showing expression of PANK4, PRDX3, NQO1 and AKR1B10 in control and PANK4-overexpressing T98G^{Res} (left) and U87MG^{Res} cells (right) at 72 and 96 hours. Tubulin was used as loading control. (**D**) Cnetplot depicting the 29 significantly downregulated proteins associated with the GO BP term “cellular response to oxidative stress”. (**E**) Gene Set Enrichment Analysis (GSEA) was performed for the GO biological process (BP) gene sets, showing enrichment of the GO “cellular response to oxidative stress” BP in siPANK4 DMSO, (**F**) siPANK4 TMZ and (**G**) siCTRL TMZ.
